# Breaking the resolution-bandwidth limit of chip-scale spectrometry by harnessing a dispersion-engineered photonic molecule

**DOI:** 10.1038/s41377-023-01102-9

**Published:** 2023-03-06

**Authors:** Hongnan Xu, Yue Qin, Gaolei Hu, Hon Ki Tsang

**Affiliations:** Department of Electronic Engineering, The Chinese University of Hong Kong, Shatin, New Territories, Hong Kong SAR, China

**Keywords:** Integrated optics, Spectrophotometry

## Abstract

The chip-scale integration of optical spectrometers may offer new opportunities for in situ bio-chemical analysis, remote sensing, and intelligent health care. The miniaturization of integrated spectrometers faces the challenge of an inherent trade-off between spectral resolutions and working bandwidths. Typically, a high resolution requires long optical paths, which in turn reduces the free-spectral range (FSR). In this paper, we propose and demonstrate a ground-breaking spectrometer design beyond the resolution-bandwidth limit. We tailor the dispersion of mode splitting in a photonic molecule to identify the spectral information at different FSRs. When tuning over a single FSR, each wavelength channel is encoded with a unique scanning trace, which enables the decorrelation over the whole bandwidth spanning multiple FSRs. Fourier analysis reveals that each left singular vector of the transmission matrix is mapped to a unique frequency component of the recorded output signal with a high sideband suppression ratio. Thus, unknown input spectra can be retrieved by solving a linear inverse problem with iterative optimizations. Experimental results demonstrate that this approach can resolve any arbitrary spectra with discrete, continuous, or hybrid features. An ultrahigh resolution of <40 pm is achieved throughout an ultrabroad bandwidth of >100 nm far exceeding the narrow FSR. An ultralarge wavelength-channel capacity of 2501 is supported by a single spatial channel within an ultrasmall footprint (≈60 × 60 μm^2^), which represents, to the best of our knowledge, the highest channel-to-footprint ratio (≈0.69 μm^−2^) and spectral-to-spatial ratio (>2501) ever demonstrated to date.

## Abstract (web summary)

Demonstration of a dispersion-engineered photonic molecule for chip-scale spectrometry beyond the resolution-bandwidth limit.

## Graphical abstract

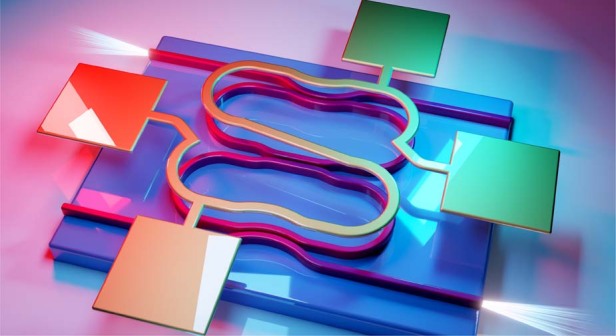

## Introduction

Spectrometry is the technology for detecting intensity information in the spectral domain. The optical spectrometer has been utilized as a potent analytical tool in many scientific and industrial applications, including but not limited to material characterization^1^, medical imaging^2^, and remote environmental monitoring^3^. Conventional benchtop spectrometers, which rely on dispersive gratings or movable components, can provide high spectral resolutions and broad working bandwidths under the laboratory condition. Nevertheless, the extensive use of benchtop spectrometers for in situ measurements is severely limited by their bulky sizes, sensitivity to platform vibration, and relatively high power consumption. There is an ever-growing demand for portable, handheld, and even wearable spectrometers in many emerging applications, such as intelligent health monitoring^4^ and portable optical coherent tomography^5^. This impetus has led to the rapid development of miniatured spectrometers implemented on metasurfaces^6^, micro-opto-electro-mechanical systems (MOEMS)^7^, and photonic integrated circuits (PIC)^8–10^. The PIC-based spectrometer has attracted tremendous research interest due to its potential for chip-scale monolithic integration. Silicon-on-insulator (SOI) is a prevalent platform for building PICs^11^. The wide transparent window of silicon, spanning from 1.1 to 8 μm, is useful in near- and mid-infrared spectroscopic sensing. The high index contrast between silicon cores and oxide claddings leads to a dramatic scale-down of device footprints. The fabrication of SOI waveguides is compatible with the CMOS technology, allowing for cost-effective and high-volume manufacturing. These immense advantages make it attractive to build high-performance spectrometers on SOI.

The spectral resolution, which quantifies the capability to resolve spectral features in fine detail, is a paramount figure of merit for spectrometers. Many applications require high resolutions throughout a broad bandwidth. For instance, the third-overtone rotational-vibrational infrared spectrum of carbon monoxide involves absorption lines with linewidths that can be <50 pm and are distributed across a wavelength range of >70 nm^12^. However, the miniaturization of spectrometers usually results in an inherent trade-off between resolutions and bandwidths. Wavelength demultiplexers, e.g., arrayed waveguide gratings (AWG)^13^ and echelle diffraction gratings (EDG)^14,15^, are commonly utilized as integrated spectrometers. Based on these schemes, the input spectrum is mapped into a point-to-point intensity distribution via spatial interferences. Therefore, a high resolution is tied to a large optical-path difference (OPD) and a small slit width. The performance of demultiplexer-based spectrometers is constrained by two factors: first, an increase in OPDs will decrease the free-spectral range (FSR) and bandwidth;^16^ and second, a reduction in slit widths will increase the crosstalk and noise^13^. For Fourier-transform spectrometers (FTS), the spectrum is reconstructed from an interferogram with a modulated time delay, thereby providing higher etendues and improved signal-to-noise ratios. Integrated FTSs can be implemented with the spatial heterodyne scheme (SHS) compromising an assembly of Mach-Zehnder interferometers (MZI) with progressively increased OPDs^17–21^. Owing to the restricted number of available MZIs, however, it is challenging to simultaneously attain high resolutions and broad bandwidths in the SHS. As an alternative, the interferogram can also be formed through the stationary-wave interference of contra-propagating beams^22–24^. This strategy is however hampered by the difficulty in the detection of stationary-wave with monolithically integrated photodetectors. Integrated speckle spectrometers, which recover spectral information from random spatial patterns, have emerged recently as a potential approach. By tailoring the disorder in scattering media^25–27^, spiral waveguides^28–31^, stratified filters^32^, or coherent networks^33^, it is possible to establish a solvable spectral-to-spatial mapping; thus, the unknown spectrum can be retrieved from the recorded pattern via computational reconstruction. In spite of this, for speckle spectrometry, it is challenging to ensure the orthogonality of patterns produced at different wavelengths.

All the aforementioned integrated spectrometers are on the basis of a static multi-channel scheme, in which the incident light is routed to a massive number of photodetectors; hence, these designs may encounter a performance bottleneck due to their large device footprints, intricate circuit topologies, and poor power efficiencies. Scanning spectrometry has the ability to circumvent these constraints by using a tunable filter to successively capture each wavelength channel. However, for most scanning spectrometers, the resolution-bandwidth limit still exists. The underlying reason is that, for most types of filters, a narrow-linewidth (i.e., resolution) is always accompanied by a narrow FSR. The measurement over multiple FSRs can be achieved by introducing an extra coarse wavelength demultiplexer^34–37^, but it is difficult to align the tuning range of filters with the wavelength grid of demultiplexers. The tandem scheme also suffers from the enlarged device footprint. Some special resonators, such as nano-beam cavities^38^ and phase-shifted gratings^39^, can offer sub-nanometer linewidths while retaining broad FSRs, thanks to the tight light confinement of photonic crystals (PhC)^40^. The paradox is that the small mode volume of PhC-based structures also restricts their tuning efficiencies, thereby limiting the tuning range. The overall bandwidth can be expanded by congregating multiple parallel filters, each of which works concurrently at a narrow tuning range^38,39^. However, the parallel scheme will increase the total footprint and aggregate heating power. The FTS can also be realized by discretely switching^41^ or continuously tuning^42–44^ the OPD of an MZI. The major drawback of integrated scanning FTSs lies in the complex switching procedure or watt-scale power consumption.

In this paper, we propose and demonstrate a ground-breaking method to overcome the resolution-bandwidth limit. We exploit the dispersive mode splitting of a photonic molecule to identify the spectral information beyond the FSR limit. As the coupled resonators are simultaneously tuned over a single FSR, a distinctive scanning trace is produced at each wavelength channel to ensure a sufficient decorrelation spanning multiple FSRs. In the Fourier domain, each left singular vector of the transmission matrix is mapped to a specific sampling frequency, making it possible to reconstruct the input spectra from the recorded signal and a characterized transmission matrix. The preconditioned least squares method is exploited to alleviate the impact of noises and handle any arbitrary spectra with discrete, continuous, or hybrid features. Experimental results demonstrate an ultrahigh resolution (<40 pm) across an ultrabroad bandwidth (>100 nm), yielding a wavelength-channel capacity of 2501. The device footprint is also as small as 60 × 60 μm^2^.

## Results

### Design principle

**Fig. 1:**
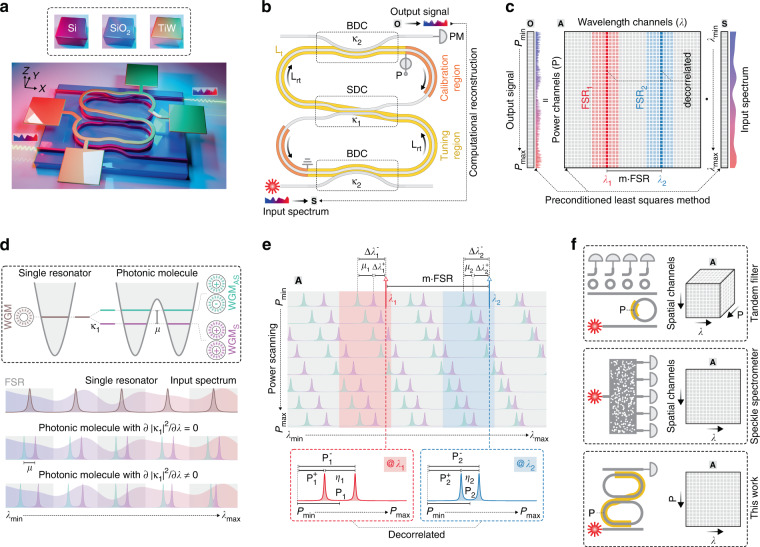
Conceptual illustration of the spectrometer. **a** 3D view of the spectrometer. **b** Schematic layout of the spectrometer. The proposed design is a photonic molecule consisting of two identical micro-ring resonators (MRR) that are synchronously tuned over a single free-spectral range (FSR). By linearly sweeping the applied heating power (*P*), the input spectrum (**S**) is scanned while the output signal (**O**) is recorded. **c** Reconstruction principle. The unknown **S** and recorded **O** are related by a transmission matrix (**A**). **S** can be reconstructed from **O** by utilizing the iterative optimization to solve a linear inverse problem if all the wavelength channels of **A** are decorrelated. **d** Dispersion engineering. For a single MRR, the transmission responses at different FSRs are indistinguishable. For a photonic molecule, the mode-splitting effect results in a symmetric mode (WGM_S_) and an anti-symmetric mode (WGM_AS_) at each FSR. If the inter-resonator coupling strength (|κ_1_|^2^) is dispersive, the splitting strength (*μ*) will vary with wavelengths. **e** Decorrelation mechanism. The scanning trace at each wavelength (*λ*_*i*_) features a pair of peaks with the mean location of *P*_*i*_ and spacing of *η*_*i*_. Owing to the dispersion of *μ*, *η*_1_, and *η*_2_ are distinct even for the wavelengths with *λ*_2_ = *λ*_1_ + *m*∙FSR (where *m* is an integer), thereby enabling the identification of each *λ*_*i*_ from *P*_*i*_ and *η*_*i*_. **f** Comparison of various types of spectrometers

Figure 1(a), (b) illustrate the schematic layout of the proposed design. The structure is a photonic molecule consisting of two micro-ring resonators (MRR) with identical round-trip lengths (*L*_rt_). The coupled resonators are controlled in a synchronized manner by an integrative thermo-optical (TO) tuning region. The functionality of the calibration region will be discussed later. By linearly sweeping the applied heating power (*P*), the input spectrum (denoted as **S**) is scanned while the output signal (denoted as **O**) is recorded. The relationship between **S** and **O** can be formulated as a set of linear equations:

1 $${{{\mathbf{O}}}} = {{{\mathbf{AS}}}}$$

where **A** denotes the transmission matrix of the spectrometer. Each column of **A** is the power scanning trace at a specific wavelength, whereas each row of **A** is the spectral transmission response at specific power. Each element of **O** is the product of the corresponding row vector and **S**, as illustrated in Fig. 1(c). The objective is to reconstruct **S** from the recorded **O** and characterized **A** by solving the linear inverse problem defined by Eq. (1). To ensure an accurate reconstruction, all the wavelength channels of **A** must be sufficiently decorrelated. The equally distributed resonances of a single MRR result in the high correlation between the wavelength channels spaced by an integral multiple of FSRs. For the photonic molecule, each whispering gallery mode splits into a symmetric mode (WGM_S_) and an anti-symmetric mode (WGM_AS_)^45^, which resembles the energy levels of a two-atom system, as illustrated in Fig. 1(d). Notably, the splitting strength (*μ*) is proportional to the inter-resonator coupling strength (|κ_1_|^2^). Thus, it is feasible to achieve a highly wavelength-dependent *μ* by enhancing the dispersion of |κ_1_|^2^. This novel property is leveraged to discriminate the wavelength channels at different FSRs, as illustrated in Fig. 1(e). Owing to the mode splitting, each wavelength is “swept” twice when tuning over a single FSR; therefore, the scanning trace at each wavelength channel (*λ*_*i*_) involves a pair of peaks. The locations of peaks (*P*^–^_*i*_, *P*^+^_*i*_) are determined by their spectral distances (Δ*λ*^−^_*i*_, Δ*λ*^+^_*i*_) to the nearest WGM_S_ and WGM_AS_. For clarity, we encode each pair of peaks with their mean locations [i.e., *P*_*i*_ = (*P*^−^_*i*_ + *P*^+^_*i*_)/2] and spacings (i.e., *η*_*i*_ = *P*^−^_*i*_ − *P*^+^_*i*_). For any two selected wavelength channels with *λ*_2_ = *λ*_1_ + *m*∙FSR (where *m* is an integer), their *P*_1_ and *P*_2_ can be equal, but theirs *η*_1_ and *η*_2_ must be different due to the dispersive splitting strengths (i.e., *μ*_1_ ≠ *μ*_2_). For other trivial cases (i.e., *λ*_2_ ≠ *λ*_1_ + *m*∙FSR), the decorrelation is ensured by the distinct *P*_1_ and *P*_2_, which is the same as for a single MRR. Consequently, each *λ*_*i*_ can be identified from an exclusive combination of *P*_*i*_ and *η*_*i*_, thereby enabling the decorrelation across an ultrabroad bandwidth far exceeding a single FSR. More details about the decorrelation mechanism can be found in Supplementary information, Section S1. Various similar schemes are compared in Fig. 1(f). For a tandem filter, the responses at different FSRs are sorted by an additional demultiplexer, necessitating multi-channel detection and complex calibration. Speckle spectrometers also rely on the computational reconstruction, but it is difficult to optimize the decorrelation property of a disordered structure with complicated topologies. In contrast, our proposed design is based solely on a simple photonic molecule, and only a single spatial channel is required.

**Fig. 2:**
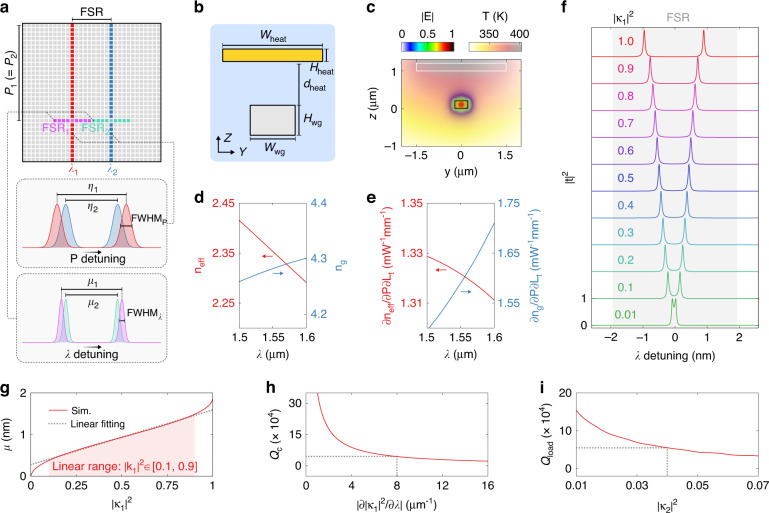
Analysis of the photonic molecule. **a** Critical condition for the wavelength-channel decorrelation. **b** Schematic cross section of the waveguide with key parameters labeled. **c** Calculated TE_0_ mode profile (|**E**|) and temperature distribution (*T*). **d** Calculated effective indices (*n*_eff_) and group indices (*n*_*g*_) of TE_0_ at varying wavelengths. **e** Calculated tuning coefficients (∂*n*_*eff*_/∂*P*∂*L*_*t*_, ∂*n*_*g*_/∂*P*∂*L*_*t*_) at varying wavelengths. **f** Calculated transmission responses (|*t*|^2^) of a photonic molecule with varying inter-resonator coupling strengths (|κ_1_|^2^). **g** Calculated splitting strengths (*μ*) with varying |κ_1_|^2^. The dashed line represents the linear fitting result. **h** Calculated critical *Q* factors (*Q*_*c*_) with varying coupling-strength dispersions (|∂|κ_1_|^2^/∂*λ*|). **i** Calculated loaded *Q* factors (*Q*_load_) with varying external coupling strengths (|κ_2_|^2^). The dashed lines indicate the optimal |∂|κ_1_|^2^/∂*λ*| and |κ_2_|^2^

The critical condition for the wavelength-channel decorrelation is that, for two wavelength channels separated by a single FSR (i.e., *λ*_2_ = *λ*_1_ + FSR), the difference between *η*_1_ and *η*_2_ must be greater than the full width at half maximum of the power scanning trace (i.e., *η*_1_ − *η*_2_ > FWHM_*P*_), as illustrated in Fig. 2(a). By assuming a uniform tuning efficiency (∂*λ*/∂*P*), an explicit equivalence of this condition can be derived as:

2 $$\mu _1 - \mu _2 > {{{\mathrm{FWHM}}}}_\lambda$$

where FWHM_*λ*_ denotes the full width at half maximum of the spectral transmission response. Next, we will analyze and optimize the photonic molecule to satisfy Eq. (2). The description about simulation methods can be found in Materials and Methods. The target bandwidth spans the wavelength range from *λ* = 1.50 μm to 1.60 μm. The design is based on an oxide-cladded SOI waveguide with core dimensions of *W*_wg_ = 450 nm and *H*_wg_ = 220 nm, as shown in Fig. 2(b). A titanium-tungsten heater (*W*_heat_ = 3 μm, *H*_heat_ = 200 nm) is placed atop the waveguide with a separation of *d*_heat_ = 1 μm. Figure 2(c) shows the calculated TE_0_ mode profile at *λ* = 1.55 μm and the temperature distribution at *P* = 50 mW. We then calculate the effective indices (*n*_eff_) and group indices (*n*_*g*_) of the TE_0_ mode, as shown in Fig. 2(d). The calculated tuning coefficients (∂*n*_eff_/∂*P*∂*L*_*t*_, ∂*n*_*g*_/∂*P*∂*L*_*t*_) are shown in Fig. 2(e). Here, *L*_*t*_ denotes the length of the tuning region. The temperature sensitivities of *n*_eff_ and *n*_*g*_ are plotted in Supplementary information, Fig S2. The round-trip length is chosen as *L*_rt_ = 150 μm to support high-*Q* resonances. In Fig. 2(f), we present the calculated single FSR transmission responses (|*t*|^2^) of a photonic molecule with varying |κ_1_|^2^. The splitting strength increases from *μ* = 0 to FSR/2 when the inter-resonator coupling strength varies from |κ_1_|^2^ = 0 to 1. Figure 2(g) shows the extracted *μ* as a function of |κ_1_|^2^. Over the linear variation range, the slope is calculated to be ∂μ/∂|κ_1_|^2^ ≈ 1.2 nm. For clarity, we define the critical *Q* factor (*Q*_*c*_) by rewriting Eq. (2) as:

3 $$Q_c = \frac{{n_gL_{rt}}}{{\left| {\frac{{\partial \mu }}{{\partial \left| {\kappa _1} \right|^2}}\frac{{\partial \left| {\kappa _1} \right|^2}}{{\partial \lambda }}\lambda } \right|}}$$

In Eq. (3), the free-spectral range is formulated as FSR = *λ*^2^/(*n*_*g*_*L*_*rt*_). In Fig. 2(h), we present the calculated *Q*_*c*_ with varying coupling-strength dispersions (|∂|κ_1_|^2^/∂*λ*|). To maximize the contrast of *μ* at neighboring FSRs, the whole linear variation range (i.e., |κ_1_|^2^ ∈ [0.1, 0.9]) is loaded onto the target bandwidth of 100 nm, yielding |∂|κ_1_|^2^/∂*λ*|≈ 8 μm^−1^. The critical *Q* factor can be then determined from Fig. 2(h) as *Q*_*c*_ ≈ 4.4 × 10^4^. Thus, it is crucial to attain a high loaded *Q* factor (*Q*_load_) that exceeds *Q*_*c*_. This can be accomplished by properly choosing the external coupling strength (|κ_2_|^2^). Figure 2(i) shows the calculated *Q*_load_ as a function of |κ_2_|^2^. Here, the propagation loss is assumed to be 2 dB/cm, which is attainable for most silicon photonic foundries. The external coupling strength is optimized to be |κ_2_|^2^ = 0.04, with the corresponding loaded *Q* factor of *Q*_load_ ≈ 5.5 × 10^4^. Such a high *Q*_load_ also allows for a high resolution. The dispersion engineering has two goals: first, the dispersion of |κ_1_|^2^ must be enhanced to cover the whole linear variation range; and second, the dispersion of |κ_2_|^2^ must be mitigated to ensure *Q*_load_ > *Q*_*c*_ across the whole bandwidth.

**Fig. 3:**
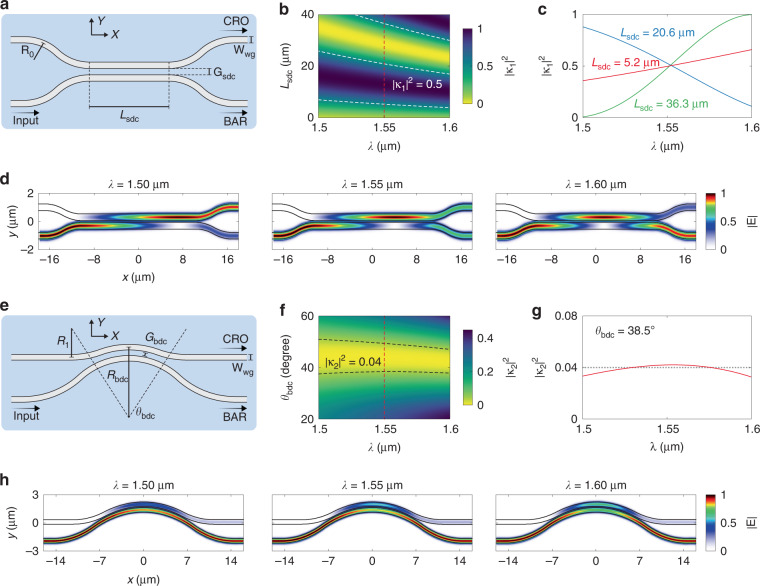
Dispersion engineering for couplers. **a** Schematic layout of the straight directional coupler (SDC) with key parameters labeled. **b** Calculated inter-resonator coupling strengths (|κ_1_|^2^) with varying coupling lengths (*L*_SDC_) and wavelengths. The white lines represent |κ_1_|^2^ = 0.5, whereas the red line indicates the central wavelength. **c** Calculated |κ_1_|^2^ with three selected *L*_SDC_. **d** Calculated light propagation profiles for SDCs. **e** Schematic layout of the bent directional coupler (BDC) with key parameters labeled. **f** Calculated external coupling strengths (|κ_2_|^2^) with varying coupling angles (*θ*_BDC_) and wavelengths. The black lines represent |κ_2_|^2^ = 0.04, whereas the red line indicates the central wavelength. **g** Calculated |κ_2_|^2^ with *θ*_BDC_ = 38.5°. The dashed line depicts the target |κ_2_|^2^. **h** Calculated light propagation profiles for BDCs

The inter-resonator coupling is supported by the straight directional coupler (SDC), as shown in Fig. 3(a). The gap width of the coupling section is set as *G*_sdc_ = 150 nm while the bending radius of the leading section is set as *R*_*0*_ = 15 μm. Figure 3(b) shows the calculated |κ_1_|^2^ with varying coupling lengths (*L*_sdc_). The 3-dB coupling can be obtained at *λ* = 1.55 μm with various *L*_sdc_. Each 3-dB point features distinct dispersion properties, as shown in Fig. 3(c). The |κ_1_|^2^ dispersion is quite weak at the critical-coupling point (*L*_sdc_ = 5.2 μm); therefore, we choose the first over-coupling point at *L*_sdc_ = 20.6 μm to enhance the dispersion. The calculated inter-resonator coupling-strength decays from |κ_1_|^2^ = 0.89 to 0.10 over the target bandwidth, which satisfies the dispersion requirement. Figure 3(d) shows the calculated light propagation profiles for the optimized SDC. The external coupling is supported by the bent directional coupler (BDC), as shown in Fig. 3(e). BDCs feature a flattened |κ_2_|^2^ dispersion since the bending-induced phase mismatch can compensate the wavelength dependence of evanescent coupling^46^. In Fig. 3(f), we present the calculated |κ_2_|^2^ with varying coupling angles (*θ*_bdc_). Other parameters are set as follows: *G*_bdc_ = 150 nm, *R*_bdc_ = 19 μm, and *R*_*1*_ = 15 μm. The coupling angle is thus optimized to be *θ*_bdc_ = 38.5° to reach the target external coupling strength of |κ_2_|^2^ = 0.04, as shown in Fig. 3(g). The variation of external coupling strengths is |κ_2_|^2^ ∈ [0.034, 0.042]. Such a weak dispersion can also be observed from the calculated light propagation profiles shown in Fig. 3(h). In Supplementary information, Fig S3, we present the tolerance analysis of SDCs and BDCs.

### Analysis of the spectrometer

**Fig. 4:**
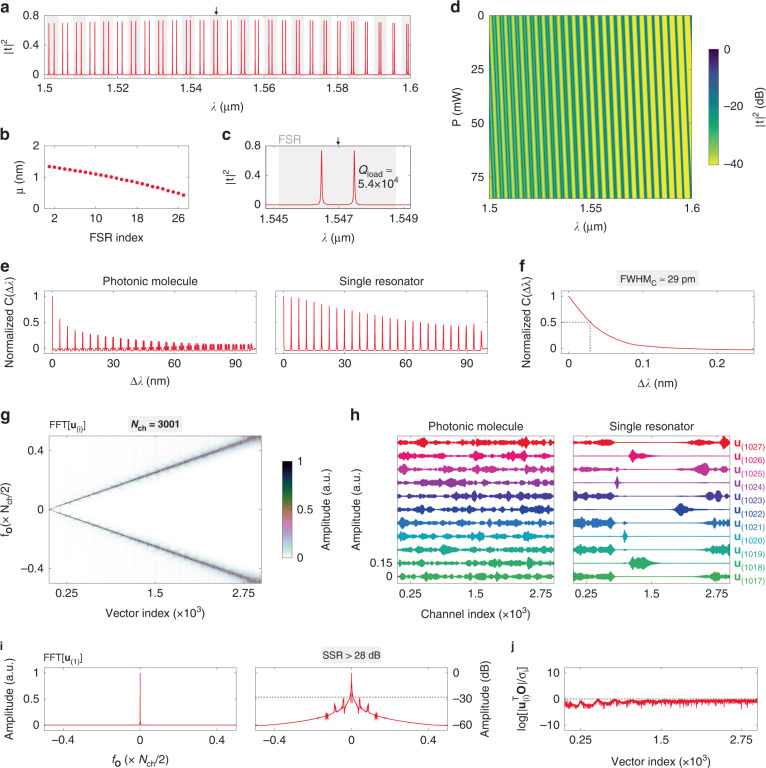
Analysis of the spectrometer. **a** Calculated transmission response (|*t*|^2^) of the optimized spectrometer with zero heating-power applied. **b** Extracted splitting strengths (*μ*) at different free-spectral ranges (FSR). **c** Enlarged view of |*t*|^2^ in the vicinity of a single FSR. **d** Calculated transmission matrix (**A**). **e** Calculated correlation functions [*C*(Δ*λ*)] for the photonic molecule (left panel) and a single resonator (right panel). **f** Enlarged view of *C*(Δ*λ*) around Δ*λ* = 0. The dashed line depicts the full width at half maximum (FWHM_*C*_). **g** Fast Fourier-transform (FFT) result for the left singular vectors [**u**_(*i*)_] of **A**. **h** Calculated **u**_(*i*)_ for the photonic molecule (left panel) and a single resonator (right panel) around the 7-th overtone of FSRs. **i** FFT result for the first left singular vector [**u**_(1)_] at the linear (left panel) and logarithmic (right panel) scales. The dashed line depicts the sideband suppression ratio (SSR). **j** Calculated absolute values of SVD coefficients [|**u**^T^_(*i*)_**O**|/σ_*i*_]. The dashed line depicts |**u**^T^_(*i*)_**O**|/σ_*i*_ = 1

On the basis of the preceding optimization results, we now present a comprehensive analysis of the spectrometer. Figure 4(a) shows the calculated |*t*|^2^ with *P* = 0 mW. The extracted *μ* at different FSRs are shown in Fig. 4(b). A strong splitting-strength contrast (*μ*_1_ − *μ*_2_ > 35 pm) at neighboring FSRs is obtained. Figure 4(c) shows the enlarged view of |*t*|^2^ in the vicinity of a single FSR. The loaded *Q* factor is estimated to be *Q*_load_ ≈ 5.4 × 10^4^ at *λ* ≈ 1.55 μm, which is in good agreement with the result shown in Fig. 2(i). In Fig. 4(d), we present the calculated transmission matrix. The length of the tuning region in each MRR is set as *L*_*t*_ = 90 μm. The heating-power variation is set as *P* = 0–85 mW to cover the required tuning range. The correlation function [*C*(Δ*λ*)] of **A** is then calculated, as shown in the left panel of Fig. 4(e). The definition of *C*(Δ*λ*) is given below:

4 $$C(\Delta \lambda ) = \left\langle {\frac{{\left\langle {I(\lambda ,P)I(\lambda + \Delta \lambda ,P)} \right\rangle _\lambda }}{{\left\langle {I(\lambda ,P)} \right\rangle _\lambda \left\langle {I(\lambda + \Delta \lambda ,P)} \right\rangle _\lambda }} - 1} \right\rangle _P$$

where *I*(*λ*,*P*) denotes the intensity at the wavelength of *λ* and heating power of *P*, <∙>_*λ*_ denotes the average over *λ*, and <∙>_*P*_ denotes the average over *P*. The displayed *C*(Δ*λ*) is normalized to *C*(0). The calculated *C*(Δ*λ*) involves a series of peaks, each of which reflects the correlation property of the wavelength channels separated by an integral multiple of FSRs. With the exception of the first peak, all the subsequent peaks decline exponentially to the low-correlation regime, which confirms that Eq. (2) is met. As a comparison, *C*(Δ*λ*) is also calculated for a single MRR with the same *L*_rt_ and |κ_2_|^2^, as shown in the right panel of Fig. 4(e) (see Supplementary information, Fig. S5 for the transmission matrix). In this instance, numerous peaks are close to *C*(Δ*λ*) ≈ 1, thereby restricting the bandwidth within a single FSR. Figure 4(f) shows the enlarged view of *C*(Δ*λ*) in the vicinity of Δ*λ* = 0. The full width at half maximum of the first peak is derived as FWHM_*C*_ ≈ 29 pm, which matches the calculated *Q*_load_. In Supplementary information, Fig. S4, we present the calculated *C*(Δ*λ*) for the spectrometer with fabrication flaws. Low inter-FSR correlation and narrow FWHM_*C*_ are maintained even with width deviations of Δ*W*_wg_ = ±20 nm, suggesting high reliability and reproducibility for different processing runs.

To perform the reconstruction, **A** must be channelized into a *N*_ch_ × *N*_ch_ square matrix with a resolution grid of *δλ* = BW/(*N*_ch_ − 1) and a scanning step of *δP* = *P*_max_/(*N*_ch_ − 1). Here, *N*_ch_ denotes the channel number, BW denotes the bandwidth, and *P*_max_ denotes the maximum heating-power applied. The channel number is chosen as *N*_ch_ = 3001 to ensure a high resolution (*δλ* ≈ 33 pm) that is close to FWHM_*C*_. More details about the channelization process can be found in Supplementary information, Section S5. Singular-value decomposition (SVD) is exploited to verify the decorrelation of wavelength channels. By utilizing SVD, **A** is transformed into the product of a diagonal matrix (**Σ**) and two sets of singular-vector spaces (**U** and **V**):^47^

5 $${{{\mathbf{A}}}} = {{{\mathbf{U}}}}{{{\mathbf{\Sigma }}}}{{{\mathbf{V}}}}^{{{\mathrm{T}}}}$$

According to Eqs. (1) and (5), the “naive” solution (**Ŝ**) to the linear inverse problem can be expressed as:

6 $${{{\hat{\mathbf S}}}} = \mathop {\sum}\limits_{i = 1}^{N_{ch}} {\frac{{{{{\mathbf{u}}}}_{(i)}^{{{\mathrm{T}}}}{{{\mathbf{O}}}}}}{{\sigma _i}}{{{\mathbf{v}}}}_{(i)}}$$

where σ_*i*_ denotes the *i*-th singular value in **Σ**, **u**_(*i*)_ denotes the *i*-th left singular vector in **U**, and **v**_(*i*)_ denotes the *i*-th right singular vector in **V**. From Eq. (6), O is “sampled” by **u**_(*i*)_ and “weighted” by σ_*i*_ to form the SVD coefficient [**u**^T^_(*i*)_**O**/σ_*i*_], which is then multiplied by the corresponding **v**_(*i*)_ to constitute **Ŝ**. If the decorrelation is sufficient, the sampling offered by **u**_(*i*)_ must be exhaustive to collect all the spectral information embedded in **O**. To demonstrate this, the fast Fourier-transform (FFT) is implemented on **u**_(*i*)_, as shown in Fig. 4(g). A straight and smooth trajectory can be observed from the FFT result, indicating a nearly point-to-point sampling on each frequency component of **O** and a complete information collection^47^. To be specific, **u**_(*i*)_ samples **O** mainly at the FFT frequency of *f*_**O**_ = *i*/2. To further prove the effectiveness of this design, we compare **u**_(*i*)_ for the photonic molecule and a single MRR with the same *L*_rt_ and |κ_2_|^2^, as shown in Fig. 4(h). As an example, we select **u**_(*i*)_ around the 7-th overtone of FSRs (i.e., *i* ≈ 7*N*_ch_FSR/BW). For the photonic molecule, **u**_(*i*)_ is a sinusoidal-like function with corrugated envelopes. For the single MRR, however, **u**_(*i*)_ becomes a pulse-like function with the majority of zero elements [see **u**_(1020)_ and **u**_(1024)_ for example]. Consequently, a substantial amount of spectral information is lost due to the insufficient sampling of **u**_(*i*)_. The similar phenomenon can be found at each overtone of FSRs, as shown in Supplementary information, Fig. S8. The underlying reason is that a single MRR is unable to recover the spectral information carried by the **O** components with *f*_**O**_ ≈ *m*∙*N*_ch_FSR/2BW (where *m* is an integer). In essence, our proposed design just aims to restore this lost portion of spectral information. Figure 4(i) shows the FFT result for the first left singular vector [**u**_(1)_]. At the logarithmic scale, the FSR-induced sidebands are significantly inhibited with a high sideband suppression ratio of SSR > 28 dB. We then plot the absolute values of SVD coefficients [|**u**^T^_(*i*)_**O**|/σ_*i*_], as shown in Fig. 4(j), to validate the solvability of the linear inverse problem. For testing purposes, **S** is set as a Gaussian function. The calculation results for other types of **S** are displayed in Supplementary information, Fig. S9. The calculated curve does not overall increase at higher vector indices, which conclusively demonstrates that, according to the Picard condition^48^, the linear inverse problem always has a convergent solution, and all the wavelength channels are solvable.

**Fig. 5:**
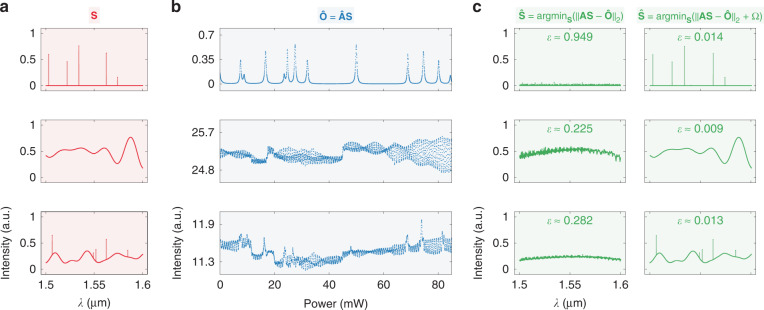
Numerical reconstruction of spectra. **a** Testing input spectra (**S**) with discrete, continuous, and hybrid features. **b** Calculated output signals (**Ȏ**). Here, **Â** denotes the transmission matrix under emulated thermo-optical perturbations. **c** Reconstructed spectra (**Ŝ**) with relative errors (*ε*) labeled. In the first and second columns, we present the reconstruction results with and without penalty terms (Ω), respectively

To further verify this design, we give a numerical example of spectrum reconstruction. Typically, there are two classes of naturally occurring spectra: discrete lines and continuous bands, as shown in the first and second rows of Fig. 5(a). In addition, the hybrid spectra with both features are also considered [see the third row of Fig. 5(a)]. In a real-world environment with noises, the measured signal (**Ȏ**) will diverge from its theoretical form:

7 $${{{\hat{\mathbf O}}}} = {{{\mathbf{O}}}} + {{{\mathbf{e}}}}$$

where **e** denotes the measurement error of **Ȏ**. For a high-*Q* resonator, the TO perturbation contributes the most to **e**^34,35^. Thus, **Ȏ** can be depicted by the equation below:

8 $${{{\hat{\mathbf O}}}} = {{{\hat{\mathbf A}\mathbf S}}} = \left( {\left[ {\begin{array}{*{20}{c}} {{{{\mathbf{a}}}}_{(1)}} \\ {{{{\mathbf{a}}}}_{(2)}} \\ \vdots \\ {{{{\mathbf{a}}}}_{(i)}} \\ \vdots \\ {{{{\mathbf{a}}}}_{(N_{ch} - 1)}} \\ {{{{\mathbf{a}}}}_{(N_{ch})}} \end{array}} \right] + \Delta {{{\mathbf{T}}}} \circ \left[ {\begin{array}{*{20}{c}} {\partial {{{\mathbf{a}}}}_{(1)}/\partial T} \\ {\partial {{{\mathbf{a}}}}_{(2)}/\partial T} \\ \vdots \\ {\partial {{{\mathbf{a}}}}_{(i)}/\partial T} \\ \vdots \\ {\partial {{{\mathbf{a}}}}_{(N_{ch} - 1)}/\partial T} \\ {\partial {{{\mathbf{a}}}}_{(N_{ch})}/\partial T} \end{array}} \right]} \right){{{\mathbf{S}}}}$$

where **Â** denotes the transmission matrix under TO perturbations, **a**_(*i*)_ denotes *i*-th row vector of **A**, Δ***T*** denotes the temperature fluctuation, and ○ denotes the Hadamard product. An integrated temperature sensor was employed to capture Δ***T***. The noise parameter can be extracted from the recorded Δ***T*** by using the Allan deviation, allowing for the emulation of actual TO perturbations. The modeling method of Δ***T*** is detailed in Supplementary information, Section S8. The calculated **Ȏ** are shown in Fig. 5(b). Owing to the existence of **e**, the linear inverse problem cannot be directly solved with Eq. (6). Instead, we choose to reconstruct **S** by using the equation below:

9 $${{{\hat{\mathbf S}}}} = \arg \mathop{\min}\limits_{{{\mathbf{S}}}}\left( {\left\| {{{{\mathbf{AS}}}} - {{{\hat{\mathbf O}}}}} \right\|_2} \right)$$

where **Ŝ** denotes the reconstructed spectrum, ||∙||_2_ denotes the $\ell_{2}$-norm, and argmin_**S**_(∙) denotes the global optimum of **S** at which the output is minimal. The iterative optimization is carried out with a least squares solver under the positivity constraint^47^. The reconstruction accuracy is quantified by the relative error (*ε*):

10 $$\varepsilon = \frac{{\left\| {{{{\mathbf{S}}}} - {{{\hat{\mathbf S}}}}} \right\|_2}}{{\left\| {{{\mathbf{S}}}} \right\|_2}}$$

The reconstruction results are shown in the first column of Fig. 5(c). It can be found that, by using Eq. (9), **Ŝ** is still severely distorted since the iterative solver tends to overfit the error-induced deviation. To tackle this problem, we modify Eq. (9) by adding penalty terms (i.e., Ω = Ω_1_ + Ω_2_):

11 $$\begin{array}{l}{{{\hat{\mathbf S}}}} = \arg \mathop{\min}\limits_{{{\mathbf{S}}}}\left( {\left\| {{{{\mathbf{AS}}}} - {{{\hat{\mathbf O}}}}} \right\|_2 + \Omega } \right)\\ \;\;\, = \arg \mathop{\min}\limits_{{{\mathbf{S}}}}\left( {\left\| {{{{\mathbf{A}}}}\left( {{{{\mathbf{S}}}}_1 + {{{\mathbf{S}}}}_2} \right) - {{{\hat{\mathbf O}}}}} \right\|_2 + \zeta _1^2\left\| {{{{\mathbf{S}}}}_1} \right\|_1 + \zeta _2^2\left\| {{{{\mathbf{D}}}}_i{{{\mathbf{S}}}}_2} \right\|_2} \right)\end{array}$$

where **S**_1_ and **S**_2_ denote the discrete and continuous components of **S**, *ζ*_1_, and *ζ*_2_ denote the regularization parameters, **D**_*i*_ denotes the *i*-th order derivative operator, and ||∙||_1_ denotes the $\ell_{1}$-norm. The first penalty term (i.e., Ω_1_ = *ζ*^2^_1_||**S**_1_||_1_) provides sparsity regularization that compresses **S**_1_ into discrete lines, whereas the second penalty term (i.e., Ω_2_ = *ζ*^2^_2_||**D**_*i*_**S**_2_||_2_) provides Tikhonov regularization that smoothens **S**_2_ into continuous bands^49^. According to Eqs. (6), (7), and (11), the reconstruction error of **Ŝ** can be derived as^47^:

12 $${{{\mathbf{S}}}} - {{{\hat{\mathbf S}}}} = \left( {{{{\mathbf{I}}}} - {{{\mathbf{V}}}}{{{\mathbf{\Psi }}}}{{{\mathbf{V}}}}^T} \right){{{\mathbf{S}}}} - {{{\mathbf{V}}}}{{{\mathbf{\Psi }}}}\mathbf \Sigma ^{ - 1}{{{\mathbf{U}}}}^{{{\mathrm{T}}}}{{{\mathbf{e}}}}$$

where **I** denotes the identity matrix, and **Ψ** denotes the filtering matrix determined by Ω. In Eq. (12), the first part [i.e., (**I**_*N*_ − **VΨV**^T^)**S**] represents the regularization penalty, whereas the second part (i.e., **VΨΣ**^−1^**U**^T^**e**) represents the noise perturbation. A proper selection of the preconditioned Ω will therefore balance the penalty and perturbation and mitigate the influence of measurement errors. The second-order derivative operator (i.e., **D**_*i*_ = **D**_2_) is employed to minimize the reconstruction error for continuous spectra while *ζ*_1_ and *ζ*_2_ are determined through the utilization of cross validation^50^. The parameter-optimization strategy is discussed in Supplementary information, Section S9. In the second column of Fig. 5(c), we present the reconstruction results after regularization. The reconstruction error is dramatically reduced to *ε* < 0.02 (see Supplementary information, Figs. S12–14 for more examples). A numerical reconstruction example of more complex spectra with stronger noises can be found in Supplementary information, Fig. S15.

### Experimental results

**Fig. 6:**
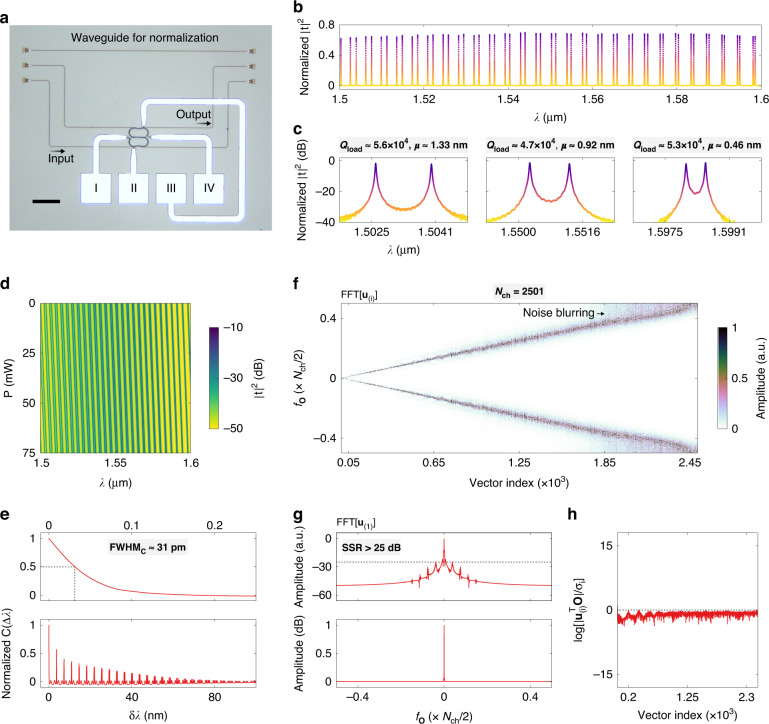
Characterization of the spectrometer. **a** Microscope image of the fabricated device. The scale bar represents 100 μm. **b** Normalized transmission response (|*t*|^2^) of the fabricated spectrometer with zero heating-power applied. **c** Enlarged views of |*t*|^2^ at three wavelength bands. **d** Measured transmission matrix (**A**). **e** Lower panel: Calculated correlation function [*C*(Δ*λ*)]. Upper panel: Enlarged view of *C*(Δ*λ*) around Δ*λ* = 0. The dashed line indicates the full width at half maximum (FWHM_*C*_). **f** Fast Fourier-transform (FFT) result for the left singular vectors [**u**_(*i*)_] of **A**. **g** FFT result for the first left singular vector [**u**_(1)_] at the linear (lower panel) and logarithmic (upper panel) scales. The dashed line depicts the sideband suppression ratio (SSR). **h** Calculated absolute values of SVD coefficients [|**u**^T^_(*i*)_**O**|/σ_*i*_]. The dashed line depicts |**u**^T^_(*i*)_**O**|/σ_*i*_ = 1

The proposed spectrometer was fabricated at a commercial silicon photonic foundry (Applied Nanotools Inc.). The microscope image of the fabricated devices is displayed in Fig. 6(a). Broadband grating couplers (GC) were employed to realize fiber-chip interconnects. A straight waveguide with GCs at both ends was fabricated in close proximity for normalization. The heater was connected to four pads (denoted as I–IV). Pads I and IV can be used to compensate the phase error resulting from fabrication flaws, as discussed in Supplementary information, Fig. S16. However, the fabricated MRRs are already in-phase, thanks to the high accuracy of electron-beam lithography (EBL); hence, only pads II and III were used in the measurement. On the same chip, some testing structures were fabricated for the characterization of SDCs and BDCs. The measurement results can be found in Supplementary information, Section S12. More details about measurement methods can be found in Materials and Methods. Figure 6(b) shows the normalized |*t*|^2^ at *P* = 0 mW. In Fig. 6(c), we present the zoom-in views of |*t*|^2^ at three wavelength bands. The measured loaded *Q* factors are *Q*_load_ > 4.7 × 10^4^. A strong dispersion of *μ* is also accomplished. The splitting contrast between adjacent FSRs is measured to be *μ*_1_ − *μ*_2_ > 33 pm, which agrees well with simulations. Figure 6(d) shows the measured **A** with the heating-power ranging from *P* = 0 mW to 75 mW. The denoising and other pre-treatments of **A** are discussed in Supplementary information, Section S5. The displayed **A** is not normalized since the non-uniform insertion loss of GCs cannot be subtracted from **Ȏ**, and the scaling of column vectors does not affect their orthogonality. The calculated *C*(Δ*λ*) of the measured **A** is shown in Fig. 6(e). The peak values drop rapidly to near zero, demonstrating that Eq. (2) is fulfilled. At the first peak, the full width at half maximum is characterized to be FWHM_*C*_ ≈ 31 pm. The Fourier analysis is then performed to validate the decorrelation, as shown in Fig. 6(f). From the FFT map, a straight and smooth trajectory can be found, suggesting that each **u**_(*i*)_ samples **Ȏ** mainly at a specific FFT frequency and a sufficient decorrelation is established. However, a few **u**_(*i*)_ are slightly blurred [see the arrow in Fig. 6(f)], which is incurred by the noise in the measured **A** and will increase the reconstruction error. Under the channel number of *N*_ch_ = 3001, it is possible to reach the theoretical resolution limit of *δλ* ≈ FWHM_*C*_ at the expense of a diminished signal-to-noise ratio of SNR < 13 dB, as can be found in Supplementary information, Fig. S19. A higher signal-to-noise ratio of SNR ≈ 20–25 dB, which is comparable to previously reported results^25,38^, can be attained by slightly reducing the channel number to *N*_ch_ = 2501, as the number of blurred **u**_(i)_ is lowered. Under the available measurement accuracy, the rigorous resolution is thus characterized to be *δλ* = 40 pm. Further analysis of noise blurring is detailed in Supplementary information, Section S13. The FFT result for **u**_(1)_ is shown in Fig. 6(g). The characterized sideband suppression ratio is SSR > 25 dB. Figure 6(h) shows the calculated |**u**^T^_(*i*)_**O**|/*σ*_*i*_ curve. The flat slope is a direct proof that the Picard condition is fulfilled and all the **u**_(*i*)_ are usable, including the blurred ones^48^. Additional Picard plots are displayed in Supplementary information, Fig. S9.

**Fig. 7:**
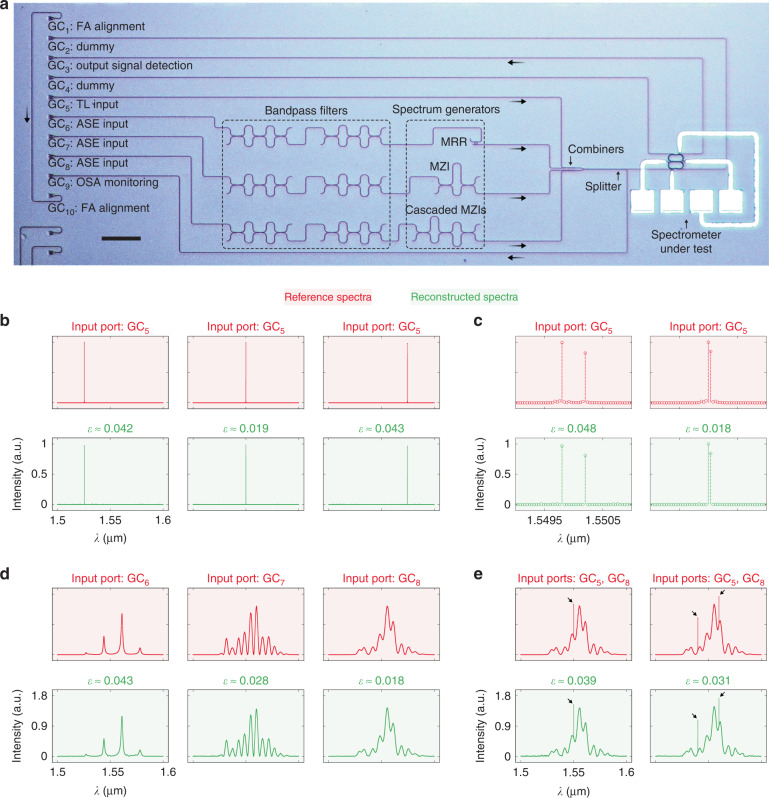
Experimental reconstruction of spectra. **a** Microscope image of the fabricated monolithic measurement system with all the on-chip components labeled. The scale bar represents 100 μm. Reconstruction results for **b** single spectral lines, **c** dual spectral lines, **d** continuous spectra, and **e** hybrid spectra with relative errors (*ε*) and used input ports labeled. Here, reference and reconstructed spectra are displayed in red and green, respectively. For clarity, dual spectral lines are displayed with zoom-in views. GC grating coupler, MRR micro-ring resonator, MZI Mach-Zehnder interferometer, TL tunable laser, ASE amplified spontaneous emission source, OSA optical spectrum analyzer, FA fiber array

A monolithic measurement system was employed to assess the experimental reconstruction of various types of spectra, as shown in Fig. 7(a). In this system, discrete spectra were produced by narrow-linewidth tunable lasers (TL), whereas continuous spectra were produced by a broadband amplified spontaneous emission (ASE) source. Distinct spectral details were loaded onto the continuous emission band with monolithically integrated filters (e.g., MRR and MZI). The out-of-range wavelengths of ASE were eliminated via bandpass filtering. Hybrid spectra with both discrete and continuous features were produced with the double injection of TL and ASE. The input spectrum was routed to the spectrometer under test and an optical spectrum analyzer (OSA), as a reference. GCs were located in an array to serve as input/output ports. The functionality of each port (denoted as GC_1_–GC_10_) is labeled in Fig. 7(a). More information about this on-chip system can be found in Supplement information, Section S14. Figure 7(b) shows the reconstruction results for single spectral lines. The reconstruction was also conducted over a wider wavelength range from *λ* = 1.50 to 1.60 μm, as shown in Supplement information, Fig. S22, demonstrating an ultrabroad bandwidth of BW = 100 nm. In Fig. 7(c), we present the reconstruction results for dual spectral lines. Two peaks can be distinguished by their different intensities, even if they were merely shifted by a single resolution grid. An ultrahigh resolution of *δλ* = 40 pm is thus demonstrated. The reconstruction results for continuous spectra are displayed in Fig. 7(d). Almost every spectral feature is properly recovered. Hybrid spectra can also be retrieved with high accuracy, as shown in Fig. 7(e). All the reconstruction results exhibit small relative errors (*ε* < 0.05).

## Discussion

In this work, we have proposed and experimentally demonstrated an integrated scanning spectrometer that breaks the resolution-bandwidth limit. By tailoring the dispersion of a photonic molecule, all the wavelength channels in the transmission matrix are decorrelated across an ultrabroad bandwidth (>100 nm) far beyond a single FSR (<4 nm). An ultrahigh resolution (<40 pm) is experimentally realized, taking advantage of the high-*Q* resonance. The spectrometer supports >2501 equivalent channels while relying solely on a single spatial channel. Through SVD and Fourier analysis, it is revealed that the dispersion-engineered photonic molecule can restore the FSR-induced information loss and collect all the spectral information from the captured signal. By utilizing the computational method to solve a linear inverse problem, it is feasible to reconstruct any arbitrary spectra with discrete, continuous, or hybrid features, even in the presence of noises.

**Fig. 8:**
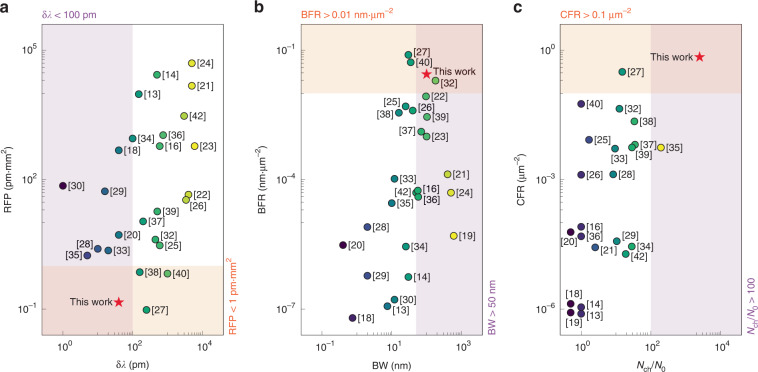
Comparison of reported spectrometers. The performance is compared with the focus on **a** resolution-footprint products (RFP) versus resolutions (*δλ*), **b** bandwidth-to-footprint ratios (BFR) versus bandwidths (BW), and **c** channel-to-footprint ratios (CFR) versus spectral-to-spatial ratios (*N*_ch_/*N*_0_)

In Supplementary information, Section S16, we present a comprehensive comparison of reported integrated spectrometers. In Fig. 8(a, b), we highlight the progress made in this work in terms of *δλ* and BW. We define the resolution-footprint product (i.e., RFP = *δλ*/*s*_*f*_) and bandwidth-to-footprint ratio (i.e., BFR = BW/*s*_*f*_) to evaluate the performance per unit footprint, given that both *δλ* and BW are related to OPD. Here, *s*_*f*_ denotes the device footprint. The proposed design is the first one that simultaneously achieves *δλ* < 100 pm and RFP < 1 pm∙mm^2^. A broad BW and a high BFR are also accomplished. In ref. ^27^, a slightly smaller RFP is realized by using an inverse-designed structure. Despite being compact, this structure can only provide a degraded resolution (*δλ* = 250 pm) and a narrow bandwidth (BW = 30 nm). The speckle spectrometer reported in ref. ^32^ also features a broad BW and a high BFR. Nevertheless, the reported resolution (*δλ* = 450 pm) is rather poor for this approach. In Fig. 8(c), we visualize the device performance regarding channel-to-footprint ratios (i.e., CFR = *N*_ch_/*s*_*f*_) and spectral-to-spatial ratios (*N*_ch_/*N*_*0*_). Here, *N*_*0*_ denotes the number of spatial channels. CFR and *N*_ch_/*N*_*0*_ reflect the capacity supported by a unit footprint and a single detector, respectively. A large channel number of *N*_ch_ > 10^4^ is obtained in ref. ^30^, in which the capacity of the FTS is boosted with aid of speckle spectrometry. The major drawback of the modified FTS is its centimeter-scale footprint and >10^5^ spatial channels. Our proposed design, in contrast, has an ultrasmall size of 60 × 60 μm^2^ with a single spatial channel, leading to the record high CFR ≈ 0.69 μm^−2^ and *N*_ch_/*N*_*0*_ > 2501. It should be noted that some applications, such as real-time optical coherence tomograph, require short acquisition time and minimal computational power. In these scenarios, multi-channel spectrometers, e.g., AWGs and EDGs, are preferable even with low CFR and *N*_ch_/*N*_*0*_ since they can simultaneously capture all wavelength channels without power scanning or computational reconstruction. It is anticipated that, by combining the proposed design with the multi-channel scheme, a relatively small *N*_*0*_ and comparatively short acquisition time can be accomplished. Specifically, the input spectrum is resolved by multiple parallel photonic molecules, each of which simultaneously scans a small number of wavelength channels. Overall, we believe that the method presented in this work will pave the way for future high-performance chip-scale spectroscopic devices.

## Materials and methods

### Simulation method

The finite-difference frequency-domain (FDFD) method is utilized to calculate the mode profile, effective indices, and group indices. For the simulation of thermo-optical tuning, the temperature distribution and tuning coefficients are obtained based on the finite element method (FEM). The transmission responses and light propagation profiles of SDCs and BDCs are calculated with the finite-difference time-domain (FDTD) method. The calculation of the transmission matrix is carried out through the co-simulation in the ANSYS Lumerical INTERCONNECT module.

### Measurement method

The transmission responses of the spectrometer were measured by using a narrow-linewidth TL (Keysight 8164B) and a synchronized PM (Agilent 81532A). A programmable power source (Keithley 2400) was employed to scan the heating power. The fabricated chip was mounted on a commercial thermo-electric cooler (TEC) to stabilize the ambient temperature. The spectrum-reconstruction setup is detailed in Supplementary information, Section S14.

## Supplementary information

Supplementary information for: Breaking the resolution-bandwidth limit of chip-scale spectrometry by harnessing a dispersion-engineered photonic molecule

## Data Availability

Data underlying this study is available from the corresponding authors upon reasonable request. Supplementary information accompanies the manuscript on the Light: Science & Applications website (http://www.nature.com/lsa).
