# Supplementary material for: Breaking the resolution-bandwidth limit of chip-scale spectrometry by harnessing a dispersion-engineered photonic molecule
Source: Light Sci Appl. 2023 Mar 6;12:64. doi: 10.1038/s41377-023-01102-9 (PMC9986235; doi:10.1038/s41377-023-01102-9)
Supplement: Supplementary file 1 — Supplementary information for: Breaking the resolution-bandwidth limit of chip-scale spectrometry by harnessing a dispersion-engineered photonic molecule [file 41377_2023_1102_MOESM1_ESM.pdf]

Supplementary information for:
Breaking the resolution-bandwidth limit of chip-scale spectrometry by harnessing a dispersion-engineered photonic molecule

Hongnan Xu*, Yue Qin, Gaolei Hu, Hon Ki Tsang*

Department of Electronic Engineering, The Chinese University of Hong Kong, Shatin, New Territories, Hong Kong SAR, China

*Corresponding Authors, E-mail: hongnanxu@cuhk.edu.hk, hktsang@ee.cuhk.edu.hk

Contents:

S1. Explanation for the wavelength-channel decorrelation

S2. Analysis of temperature sensitivity

S3. Analysis of fabrication tolerance

S4. Transmission matrix of a single micro-ring resonator

S5. Channelization of the transmission matrix

S6. Analysis of left singular vectors

S7. Picard plots

S8. Characterization of temperature fluctuations

S9. Selection of hyperparameters

S10. Additional numerical reconstruction examples

S11. Additional information about the calibration process

S12. Characterization of dispersion-engineered couplers

S13. Analysis of noise blurring

S14. Additional information about the monolithic measurement system

S15. Additional experimental reconstruction results for single spectral lines

S16. Performance comparison of reported integrated spectrometers

S1. Explanation for the wavelength-channel decorrelation

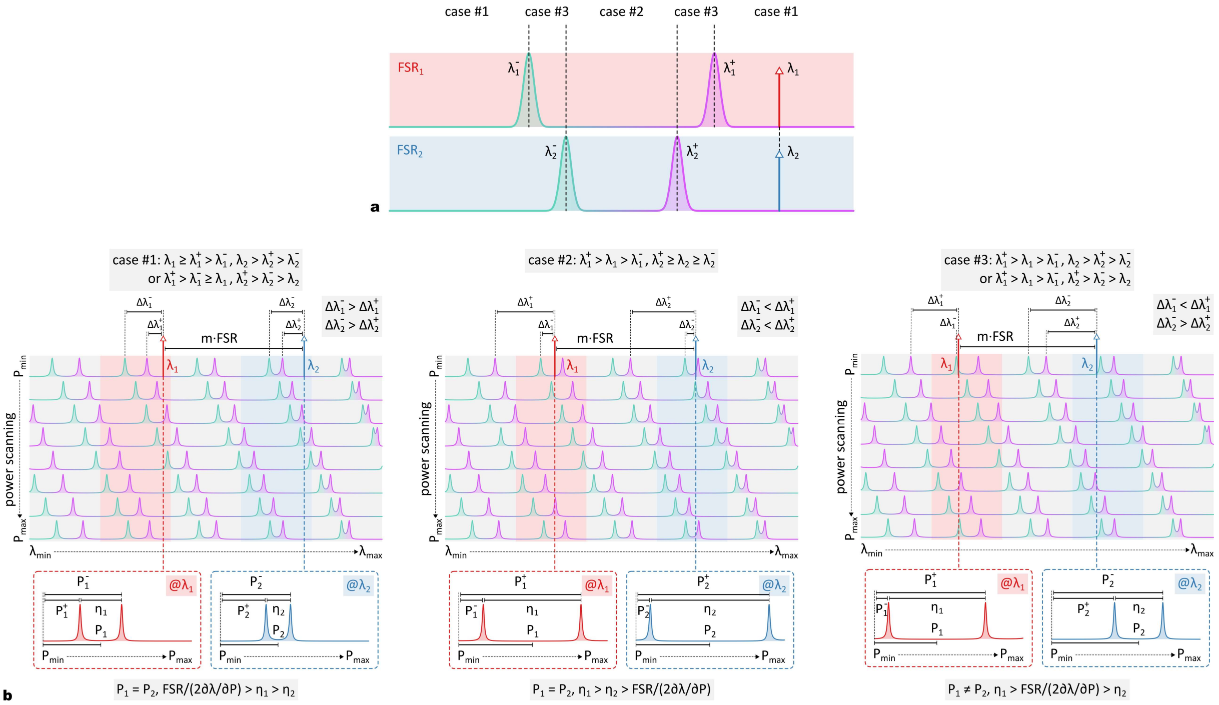

Fig. S1 Wavelength-channel decorrelation. (**a**) Illustration of wavelength channels at different free spectral ranges (FSR_1, FSR_2). For clarity, FSR_1 and FSR_2 are superimposed. (**b**) Illustration of the decorrelation mechanism.

Due to the dispersive mode splitting in the photonic molecule, each wavelength channel will be "swept" twice and encoded by its distinguishable response. Each scanning trace features a pair of peaks, with their locations related to the spectral distance ($\Delta\lambda_i^-$, $\Delta\lambda_i^+$) to the nearest resonant modes. In this design, it is of paramount importance to decorrelate the wavelength channels that are separated by an integral multiple of free spectral ranges (FSR), i.e., $\lambda_2 = \lambda_1 + m\cdot\mathrm{FSR}$ (where m is an integer), since other wavelength channels are naturally decorrelated. The situation can be classified into three different cases with respect to the contrast between $\Delta\lambda_i^-$ and $\Delta\lambda_i^+$, as illustrated in Fig. S1(a). In Fig. S1(b), we visualize the decorrelation mechanism for all three situations, which can also be expressed by the equation below:

$$\begin{cases} P_1 = P_2, \mathrm{FSR}/\left(2\partial\lambda/\partial P\right) > \eta_1 > \eta_2, & \Delta\lambda_1^- > \Delta\lambda_1^+, \Delta\lambda_2^- > \Delta\lambda_2^+ \\ P_1 = P_2, \eta_1 > \eta_2 > \mathrm{FSR}/\left(2\partial\lambda/\partial P\right), & \Delta\lambda_1^- < \Delta\lambda_1^+, \Delta\lambda_2^- < \Delta\lambda_2^+, \\ P_1 \neq P_2, \eta_1 > \mathrm{FSR}/\left(2\partial\lambda/\partial P\right) > \eta_2, & \Delta\lambda_1^- < \Delta\lambda_1^+, \Delta\lambda_2^- > \Delta\lambda_2^+ \end{cases} \tag{S1}$$

where P_1 and P_2 denote the peak locations in the scanning trace, η_1 and η_2 denote the spacings between peaks, and $\partial\lambda/\partial P$ denotes the tuning efficiency. The decorrelation for other trivial cases (i.e., $\lambda_2 \neq \lambda_1 + m\cdot\mathrm{FSR}$) is ensured by the distinct P_i. Consequently, each λ_i is encoded by a unique combination of P_i and η_i, leading to the decorrelation spanning multiple FSRs.

S2. Analysis of temperature sensitivity

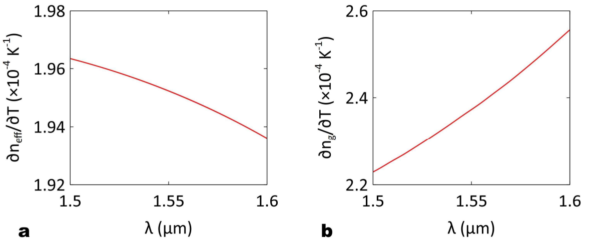

Fig. S2 Temperature sensitivity. Calculated thermo-optical (TO) coefficients of (**a**) effective indices ($\partial n_{eff}/\partial T$) and (**a**) group indices ($\partial n_g/\partial T$). At the central wavelength of λ = 1.55 μm, the TO coefficients are calculated to be $\partial n_{eff}/\partial T = 1.95\times10^{-4}$ K^{-1} and $\partial n_g/\partial T = 2.37\times10^{-4}$ K^{-1}. These simulation results are used in the emulation of TO perturbations, as discussed in Fig. 5.

S3. Analysis of fabrication tolerance

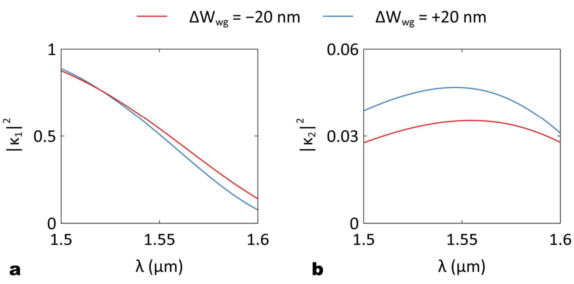

Fig. S3 Fabrication tolerance of couplers. Calculated (**a**) inter-resonator coupling strengths ($|\kappa_1|^2$) and (**b**) external coupling strengths ($|\kappa_2|^2$) with deviated waveguide widths (W_{wg}). Here, the gap width changes in accordance with the ΔW_{wg}. The $|\kappa_1|^2$ and $|\kappa_2|^2$ variations are quite minor even under fabrication flaws.

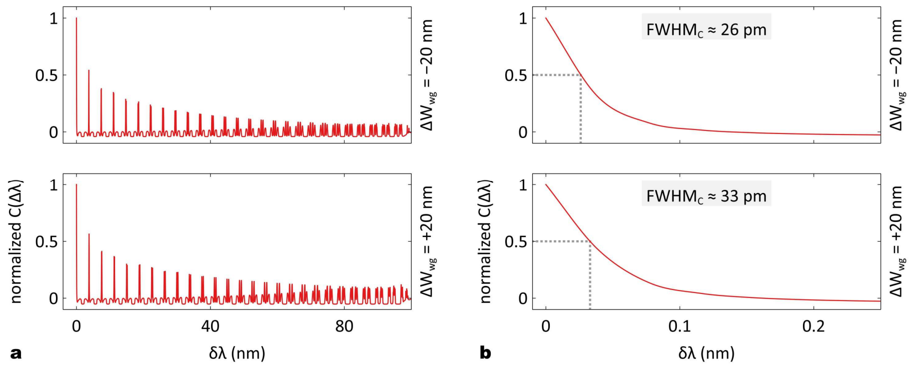

Fig. S4 Fabrication tolerance of the spectrometer. (**a**) Calculated correlation functions [$C(\delta\lambda)$] for the spectrometer with width deviations of $\delta W_{wg} = \pm 20$ nm. (**b**) Enlarged view of $C(\Delta\lambda)$ around $\Delta\lambda = 0$ with $\delta W_{wg} = \pm 20$ nm. Here, we present the tolerance analysis of the spectrometer. The deviation of waveguide widths (δW_{wg}) is the primary source of fabrication defects. From Fig. S4(a), the peaks in $C(\Delta\lambda)$ fall rapidly into the low-correlation regime, even with $\delta W_{wg} = \pm 20$ nm, indicating that width deviations will not affect the decorrelation property. The resolution limit is determined by the full width at half maximum (FWHM$_C$) of the first peak in $C(\Delta\lambda)$. It can be found from Fig. S4(b) that, with $\delta W_{wg} = \pm 20$ nm, high resolutions of FWHM$_C$ = 26 ~ 33 pm can still be attained and the loaded Q factor (Q_{load}) is still higher than the critical value [Q_c, see also Eq. (3)], demonstrating a high resolution and sufficient decorrelation. These results proves that the proposed design is robust against fabrication errors and is reproducible throughout different processing runs.

S4. Transmission matrix of a single micro-ring resonator

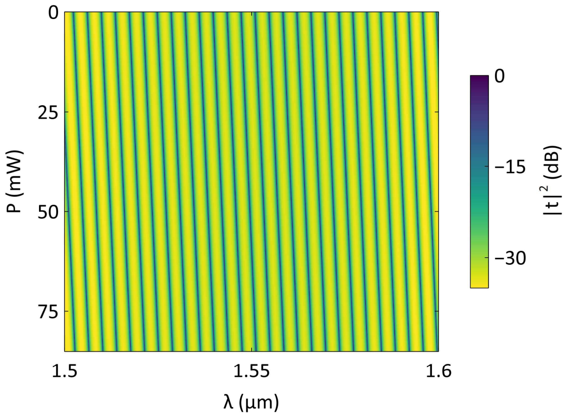

Fig. S5 Single-resonator transmission. Here, we present the calculated transmission matrix (**A**) of a single micro-ring resonator with the same round-trip length (L_{rt} = 150 μm) and external coupling strength ($|\kappa_2|^2 = 0.04$) as the photonic molecule. The calculated **A** is used in the derivation of correlation functions shown in Fig. 4(e) and left singular vectors [$\mathbf{u}_{(i)}$] shown in Figs. 4(h) and S8.

S5. Channelization of the transmission matrix

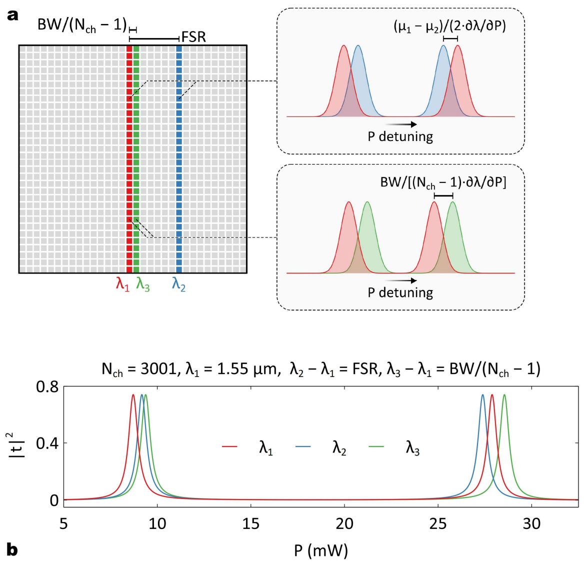

Fig. S6 Channelization mechanism. (**a**) Decorrelation of the wavelength channels spaced by a FSR (i.e., $\lambda_2 = \lambda_1 + \text{FSR}$) and a single resolution grid (i.e., $\lambda_3 = \lambda_1 + \delta\lambda$). (**b**) Calculated scanning traces at three selected wavelength channels.

The channelization mechanism is illustrated in Fig. S6(a). For the case when $\lambda_2 = \lambda_1 + \text{FSR}$, the peak spacing is $(\mu_1 - \mu_2)/(2\cdot\partial\lambda/\partial P)$. Here, μ_1 and μ_2 denote the splitting strengths, and $\partial\lambda/\partial P$ denotes the tuning efficiency. Thus, the decorrelation between these wavelength channels is guaranteed by the dispersion of μ. For the case when $\lambda_3 = \lambda_1 + \delta\lambda$, the peak spacing is $\text{BW}/[(N_{ch} - 1)\cdot\partial\lambda/\partial P]$. Here, BW denotes the working bandwidth, and N_{ch} denotes the channel number. Consequently, N_{ch} must be reduced to $\approx \text{BW}/\text{FHWM}_C$ to ensure a sufficient decorrelation between neighboring wavelength channels as well as a high resolution defined by FHWM_C. Here, FHWM_C denotes the full width at half maximum of the correlation function. An oversized N_{ch} cannot truly improve the resolution but rather increase the reconstruction error. In Fig. S6(b), we present the calculated scanning traces under the optimal channel number of $N_{ch} = 3001$. The peaks are well dislocated by more than a full width at half maximum for both $\lambda_2 = \lambda_1 + \text{FSR}$ and $\lambda_3 = \lambda_1 + \delta\lambda$.

Next, we will discuss the pre-treatments used in the channelization of the measured transmission matrix (**A**). At the *i*-th scanning step, the rigorous expression of the recorded signal (O_i) can be derived from Eq. (1) as:

$$O_i = \int_{\lambda_{min}}^{\lambda_{max}} \mathbf{a}_{(i)} \mathbf{S} d\lambda = \sum_{j=1}^{N_{ch}} \int_{\lambda_j - \delta\lambda/2}^{\lambda_j + \delta\lambda/2} \mathbf{a}_{(i)} \mathbf{S} d\lambda, \tag{S2}$$

where $\mathbf{a}_{(i)}$ denotes the *i*-th row vector in **A**, and **S** denotes an unknown spectrum to be resolved. One straightforward channelization approach is to directly pick elements from the initial row vector [see Fig. S7(a) and S7(b)]:

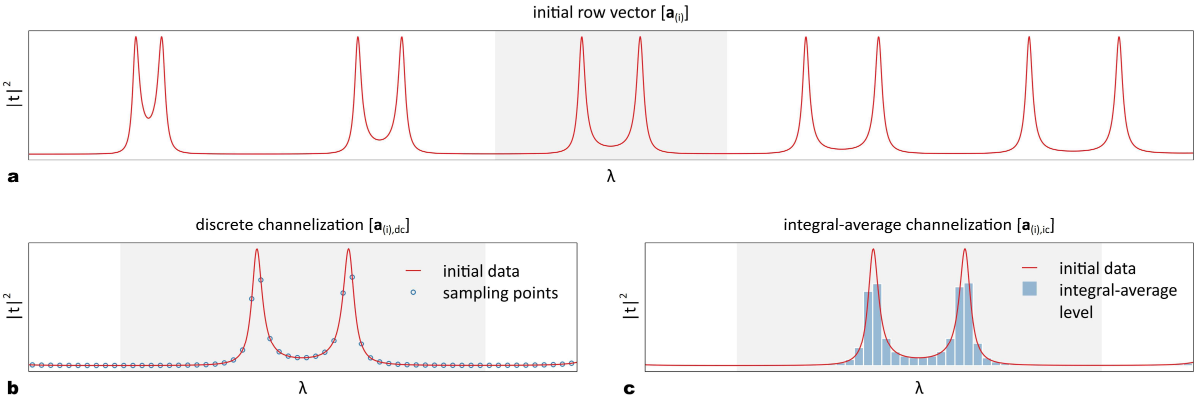

Fig. S7 Channelization method. (**a**) Initial row vector before channelization. Illustration of (**b**) discrete and (**c**) integral-average channelization methods.

$$a_{ij,dc} = \mathbf{a}_{(i)}\big|_{\lambda=\lambda_j}, \tag{S3}$$

where $a_{ij,dc}$ denotes the j-th element in the channelized row vector $[\mathbf{a}_{(i),dc}]$. This method can offer high accuracy for the reconstruction of discrete spectra since the linewidth of the used tunable laser is much narrower than δλ. Also, we used the same tunable laser in the characterization of **A** [see Fig. 6(d)] and production of spectral lines [see Fig. 7(b-c)], which means that, by using Eq. (S3), the recorded signal will be in good accord with its theoretical form. However, the scenario is different for the reconstruction of continuous spectra since, around resonant wavelengths, the $\mathbf{a}_{(i)}$ intensity varies significantly over δλ. By assuming the slow variation of **S**, Eq. (S2) can be rewritten as:

$$O_i \approx \sum_{j=1}^{N_{ch}} \left(\frac{1}{\delta\lambda} \int_{\lambda_j - \delta\lambda/2}^{\lambda_j + \delta\lambda/2} \mathbf{S} d\lambda \right) \int_{\lambda_j - \delta\lambda/2}^{\lambda_j + \delta\lambda/2} \mathbf{a}_{(i)} d\lambda. \tag{S4}$$

Noticing that the j-th element in **S** is the integral over a single resolution grid (see the term in brackets), we utilize the integral-average channelization to handle continuous spectra [see Fig. S7(a) and S7(c)], as can be formulated by the equation below:

$$a_{ij,ic} = \frac{1}{\delta\lambda} \int_{\lambda_j - \delta\lambda/2}^{\lambda_j + \delta\lambda/2} \mathbf{a}_{(i)} d\lambda, \tag{S5}$$

where $a_{ij,ic}$ denotes the j-th element in the channelized row vector $[\mathbf{a}_{(i),ic}]$.

For simplicity, all the numerical results are based on the discrete channelization defined by Eq. (S3). In addition, for correlation functions, SVD analysis, and Picard plots, the measured **A** is also channelized by utilizing Eq. (S3). For the experimental reconstruction of spectra, however, it is necessary to consider both discrete and continuous components of **S**. Thus, from Eqs. (S3) and (S5), Eq. (11) is modified as follows:

$$\hat{\mathbf{S}} = \arg\min_{\mathbf{S}} \left(\left\| \mathbf{A}_{dc}\mathbf{S}_1 + \mathbf{A}_{ic}\mathbf{S}_2 - \hat{\mathbf{O}} \right\|_2 + \zeta_1^2 \left\| \mathbf{S}_1 \right\|_1 + \zeta_2^2 \left\| \mathbf{D}_i \mathbf{S}_2 \right\|_2 \right), \tag{S6}$$

where $\mathbf{A}_{dc}$ and $\mathbf{A}_{ic}$ are the transmission matrices under discrete and integral-average channelization, respectively. The definitions of other parameters can be found in the main manuscript. Before channelization, the measured **A** is first denoised. The raw data involves $\approx 100 \cdot N_{ch}$ points at each scanning step. The stochastic noise in $\mathbf{a}_{(i)}$ is filtered out by applying a sliding window over 25 points with the weighted polynomial regression at the logarithmic scale[S1]. The local regression is error-free since the ideal response of a micro-ring resonator is fairly smooth in at logarithmic scale and the responses are well within the dynamic range (≈ 80 dBm) of the optical power meter. After denoising, high-frequency noises are inhibited to an ultralow level. Nevertheless, a small portion of low-frequency noises that are out of the sliding window still exist, resulting in the blurring effect shown in Fig. 6(f). The measured **A** is not normalized in the SVD analysis and spectrum reconstruction [see Figs. 6(f)-6(h) and 7], whereas in the calculation of correlation functions [see Fig. 6(e)], the insertion loss of grating couplers is deducted.

S6. Analysis of left singular vectors

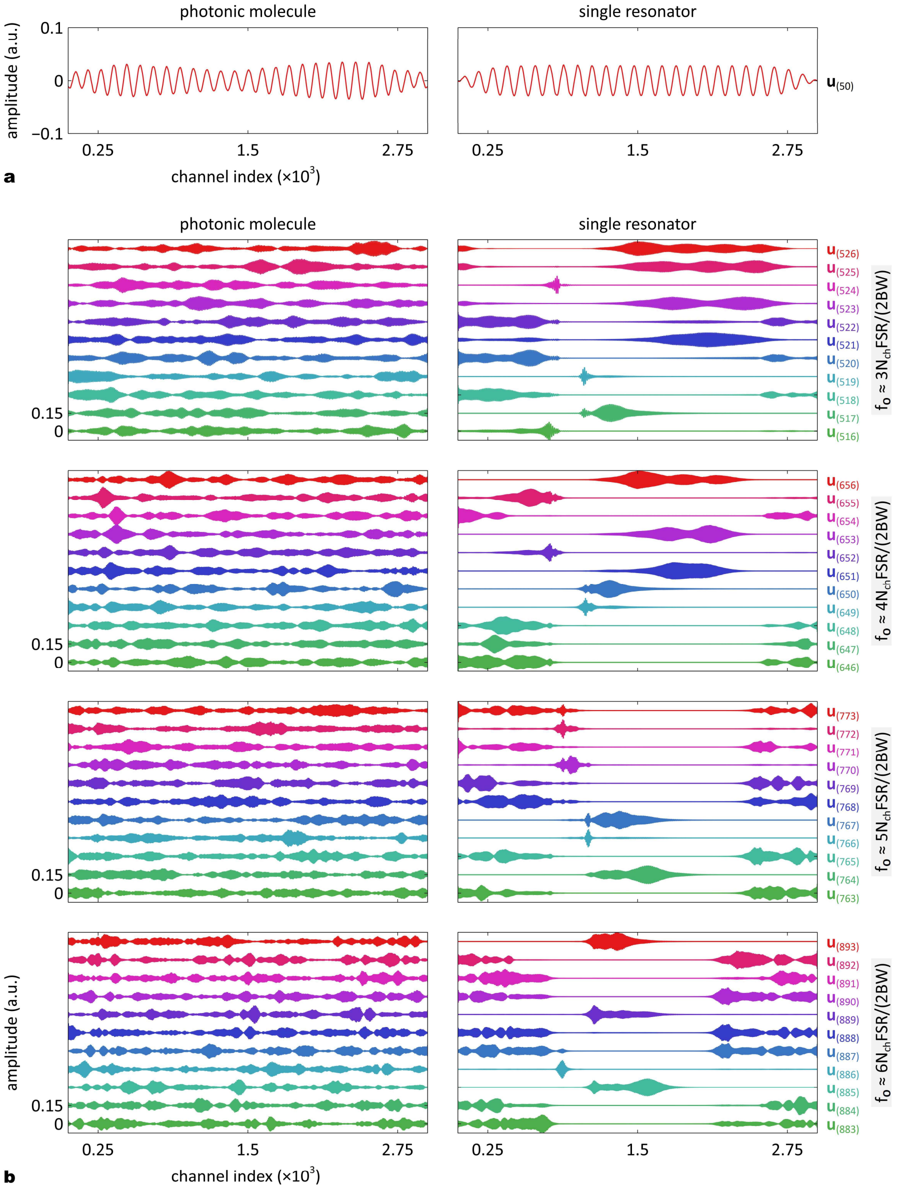

Fig. S8 Singular-vector analysis. (**a**) Calculated 50-th left singular vector [$\mathbf{u}_{(50)}$] for the photonic molecule (left panel) and a single resonator (right panel) with the same round-trip length and external coupling strength. Here, the selected vector index is within the first overtone of FSRs (i.e., $50 < N_{ch}$FSR/BW). It can be found that, for both cases, $\mathbf{u}_{(50)}$ is a sinusoidal-like function with a virtually uniform envelope. (**b**) Calculated left singular vectors [$\mathbf{u}_{(i)}$] for the third to sixth overtone of FSRs (i.e., $i \approx m{\cdot}N_{ch}$FSR/BW, m = 3~6). The FFT frequency of $\mathbf{u}_{(i)}$, which is also the sampling frequency on the output signal (**O**), increases at higher indices. For the photonic molecule, the calculated $\mathbf{u}_{(i)}$ maintains as a sinusoidal-like function, with its envelope slightly corrugated. For the single resonator, however, the calculated $\mathbf{u}_{(i)}$ becomes a pulse-like function that is dominated by zero elements [see $\mathbf{u}_{(519)}$, $\mathbf{u}_{(649)}$, $\mathbf{u}_{(766)}$, and $\mathbf{u}_{(886)}$ for example]. This reflects the fact that a single resonator is incapable of handling the spectral information conveyed by the **O** component with the FFT frequency of $f_{\mathbf{O}} = m{\cdot}N_{ch}$FSR/2BW (where m is an integer). By leveraging the proposed photonic molecule, all the wavelength channels are sufficiently decorrelated even for those separated by an integral multiple of FSRs, thereby restoring the lost spectral information.

S7. Picard plots

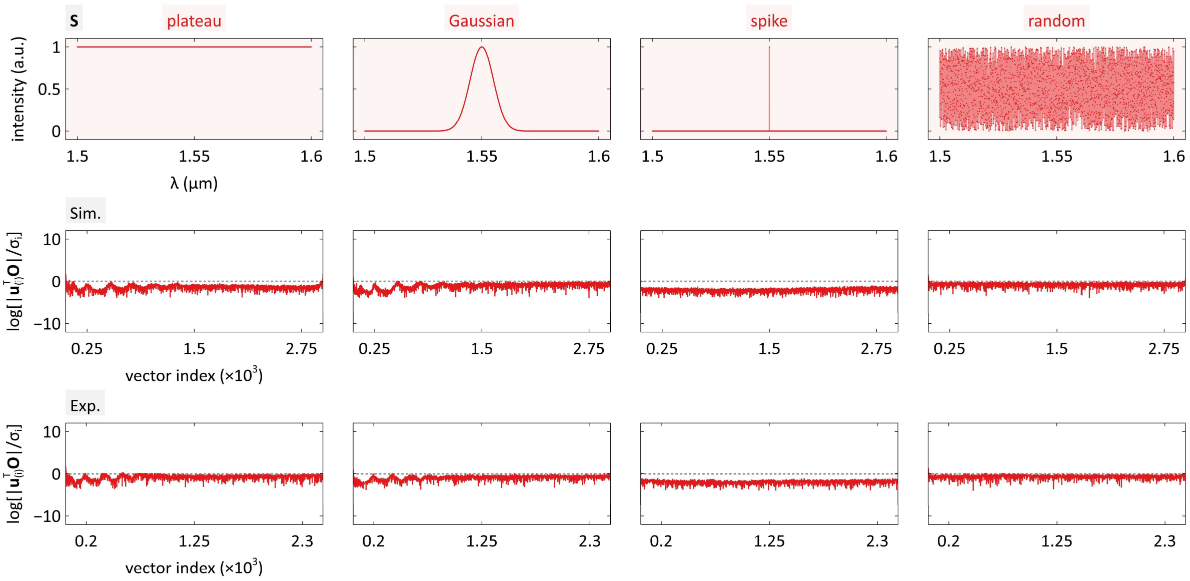

Fig. S9 Picard plots. The first row shows the generated input spectra (**S**). Here, plateau, Gaussian, spike, and random functions with radically different features are employed for testing purposes. The second row shows the calculated absolute values of SVD coefficients ($|\mathbf{u}_{(i)}^{T}\mathbf{O}|/\sigma_i$), also known as the Picard plot. The third row shows the Picard plots for experimental results. The dashed lines represent $|\mathbf{u}_{(i)}^{T}\mathbf{O}|/\sigma_i = 1$. The calculated curves all remain below $|\mathbf{u}_{(i)}^{T}\mathbf{O}|/\sigma_i < 1$, indicating that the decay rate of σ_i does not exceed that of $|\mathbf{u}_{(i)}^{T}\mathbf{O}|$, and that the SVD coefficient has a finite integral. According to the Picard condition[S2], this is a direct proof that the linear inverse problem always has a convergent solution, and that all the wavelength channels are solvable. The Picard plots for the Gaussian function are also displayed in Figs. 4(j) and 7(h).

S8. Characterization of temperature fluctuations

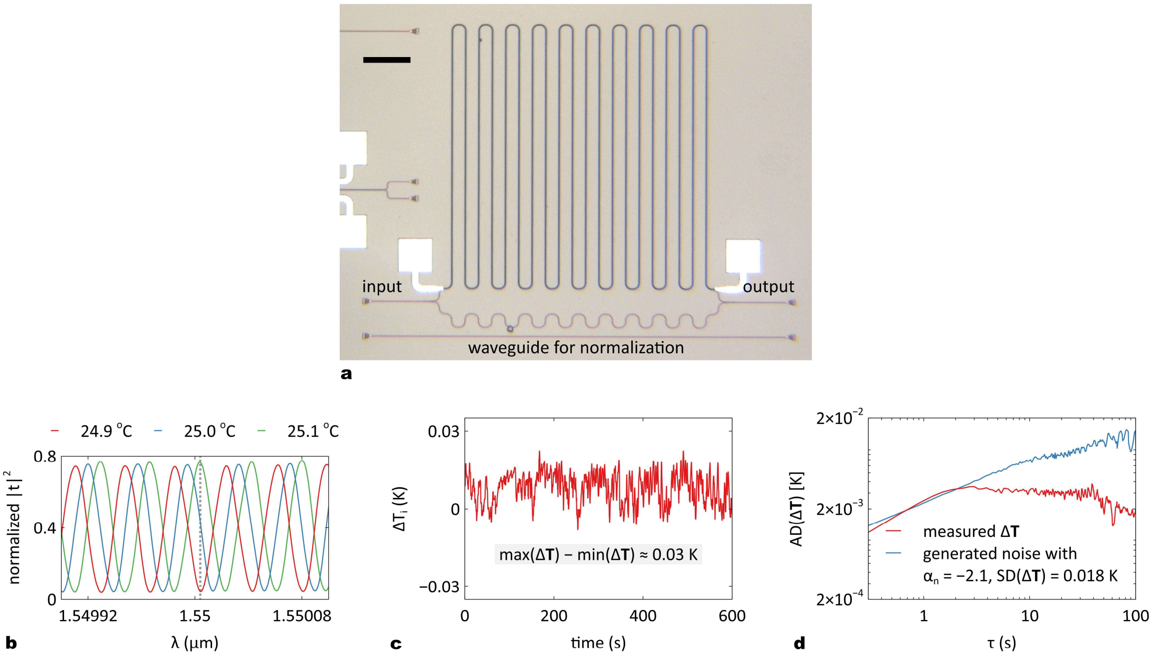

Fig. S10 Temperature measurement. (**a**) Microscope image of the fabricated temperature sensor. (**b**) Normalized transmission responses ($|t|^2$) at different temperatures. (**c**) Extracted temperature fluctuations (Δ**T**) as a time sequence. (**d**) Calculated Allen deviations (AD) of Δ**T** and the generated noise with the fitted noise parameter (α_n) and standard deviation (SD).

An integrated temperature sensor was employed to capture the temperature fluctuation (Δ**T**) in the measurement system. The device is a Mach-Zehnder interferometer consisting of a 1.5-cm phase shifter and two 3-dB couplers, as shown in Fig. S10(a). Figure S10(b) shows the normalized transmission responses ($|t|^2$) at different temperatures. The measurement wavelength was fixed at $\lambda \approx 1.55$ μm. The output power was recorded as a time sequence so that Δ**T** can be extracted from the collected single-channel signal, as shown in Fig. S10(c). Here, the acquisition time at each sampling point is ≈ 0.3 s, which is the same as for the scanning procedure applied in the spectrum detection. The noise parameter (α_n) and standard deviation (SD) are two key parameters in the modeling of Δ**T**. To be specific, α_n reflects the distribution of power spectral densities (i.e., log[PSD(Δ**T**)] ~ α_n) whereas SD(Δ**T**) reflects the noise strength. The Allan deviation (AD) is utilized to derive α_n and SD, as shown in Fig. S10(d). The definition of AD is given below[S3]:

$$\mathrm{AD}(\Delta\mathbf{T}) = \sqrt{\frac{1}{2}\left\langle \left(\Gamma_{i+1} - \Gamma_i\right)^2 \right\rangle}, \tag{S7}$$

where Γ_i denotes the i-th fractional frequency average of Δ**T** over a truncated time window of τ, and <·> denotes the average over fractional frequencies. In a log-log plot, α_n and SD can be determined from the slope and intercept of the curve[S1]. By fitting the obtained AD(Δ**T**) with the generated noise, the noise parameter and standard deviation

are estimated to be $\alpha_n = -2.1$ and $SD(\Delta\mathbf{T}) = 0.018$ K, respectively. Such a fluctuation level can be realized by most commercial thermo-electric coolers (TEC). The divergence of curves at the large τ results from the limited number of sampling points[S3]. Notably, the measured $\Delta\mathbf{T}$ is a Brownian noise ($\alpha_n \approx -2$) rather than a white noise ($\alpha_n \approx 0$)[S1]. This property is governed by the feedback mechanism of the TEC. A fully stochastic $\Delta\mathbf{T}$ with the characterized α_n and AD is generated and applied in each numerical example shown in Figs. 5 and S11-S14.

S9. Selection of hyperparameters

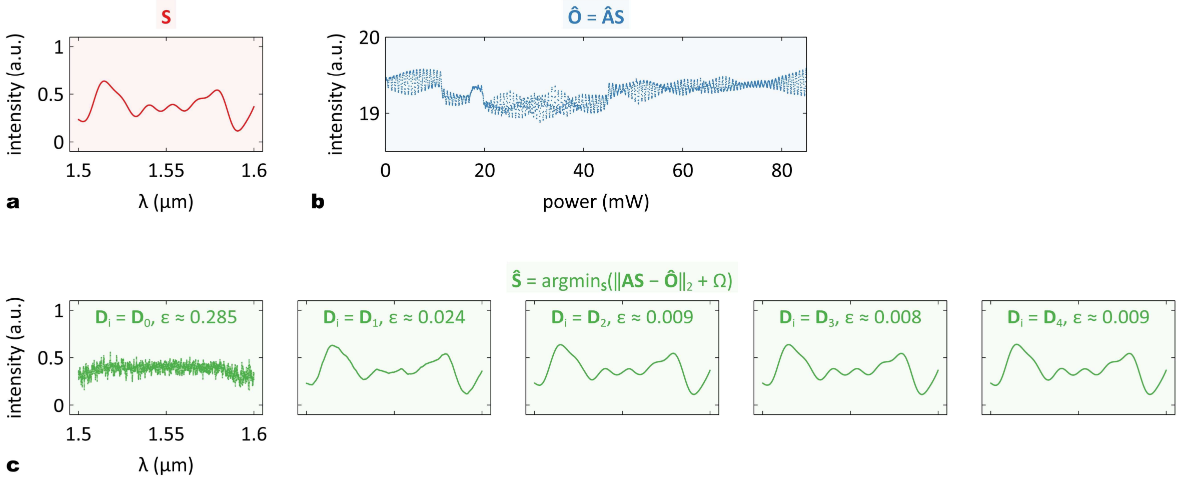

Fig. S11 Selection of the derivative operator. (**a**) Testing input spectrum (**S**). (**b**) Calculated output signal ($\hat{\mathbf{O}}$). (**c**) Reconstructed spectra ($\hat{\mathbf{S}}$) with relative errors (ε) labeled.

Some hyperparameters (i.e., ζ_1, ζ_2, and $\mathbf{D}_i$) must be optimized to accomplish the balance between the regularization penalty and noise perturbation [see Eq. (12)]. In this work, ζ_1 and ζ_2 are determined through cross validation (CV)[S4]. The concept of CV is to divide $\hat{\mathbf{O}}$ into two sets, one of which is utilized to generate a "reduced" solution to predict the elements in the other set. The prediction error reaches its minimum with the optimal ζ_1 and ζ_2:

$$(\zeta_1,\zeta_2) = \arg\min_{\zeta_1,\zeta_2}\left[\frac{1}{N_{ch}}\sum_{i=1}^{N_{ch}}\left(\mathbf{a}_{(i)}\hat{\mathbf{S}}_{(i)} - O_i\right)^2\right], \tag{S8}$$

where N_{ch} denotes the channel number, $\mathbf{a}_{(i)}$ denotes the i-th row vector of the transmission matrix ($\mathbf{A}$), $\hat{\mathbf{S}}_{(i)}$ denotes the solution with $\mathbf{a}_{(i)}$ left out in $\mathbf{A}$, O_i denotes the i-th element of $\hat{\mathbf{O}}$, and $\arg\min_{\zeta_1,\zeta_2}(\cdot)$ denotes the global minimum. However, it is time-consuming to implement the standard CV since it requires N_{ch} rounds of regularization at each iteration. The search of the optimal ζ_1 and ζ_2 can be accelerated by using generalized, K-fold, or rolling CV, which can be supported by many open-source tools, such as LASSOPACK[S5]. Here, ζ_1 and ζ_2 are optimized by exploiting K-fold CV with $K = 10$. The priori knowledge of spectral features also helps to accelerate the process. For instance, if we already know that the spectrum solely contains discrete spectral lines, then ζ_2 can be set as zero and only ζ_1 needs to be optimized. Notably, due to the great convergence validated by the Picard plot (see Fig. S9), it is feasible to attain the same level of reconstruction accuracy without any priori. In other words, ζ_1/ζ_2 will automatically drop to near zero for continuous/discrete spectra during the CV procedure, and the presetting of hyperparameters is only for shortening the optimization time. The further discussion on this issue can be found in Fig. S15. In Fig. S10, we present a numerical test for the reconstruction of continuous spectra under $\mathbf{D}_i$ of different orders. A minimal error of $\varepsilon < 0.01$ is attained with the second-order derivative operator (i.e., $\mathbf{D}_i = \mathbf{D}_2$), which is thus used in the numerical

and experimental reconstruction. The expression of $\mathbf{D}_2$ is as follows:

$$\mathbf{D}_2 = \begin{bmatrix} 1 & -2 & 1 & & \\ & \ddots & \ddots & \ddots & \\ & & 1 & -2 & 1 \end{bmatrix} \in \mathbb{R}^{(N_{ch}-2)\times N_{ch}}. \tag{S9}$$

S10. Additional numerical reconstruction examples

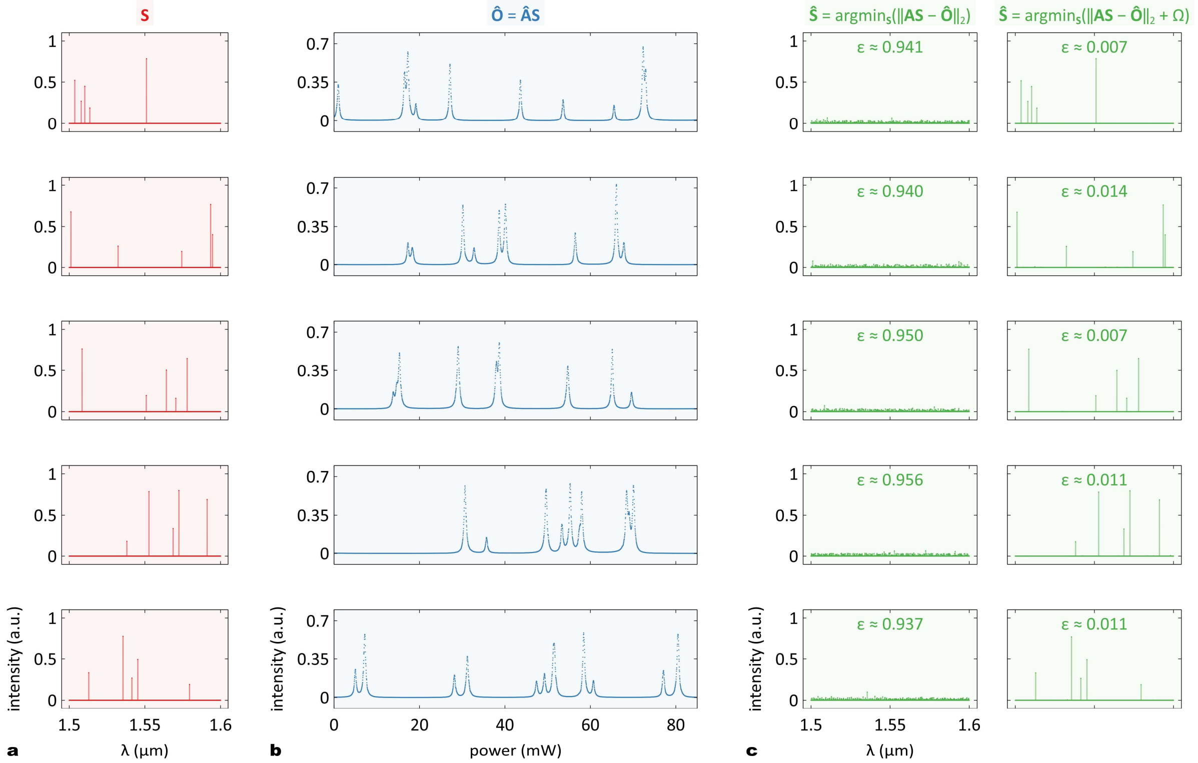

Fig. S12 Reconstruction of discrete spectra. (**a**) Testing input spectra (**S**). (**b**) Calculated output signals (**Ô**). (**c**) Reconstructed spectra (**Ŝ**) with relative errors (ε) labeled.

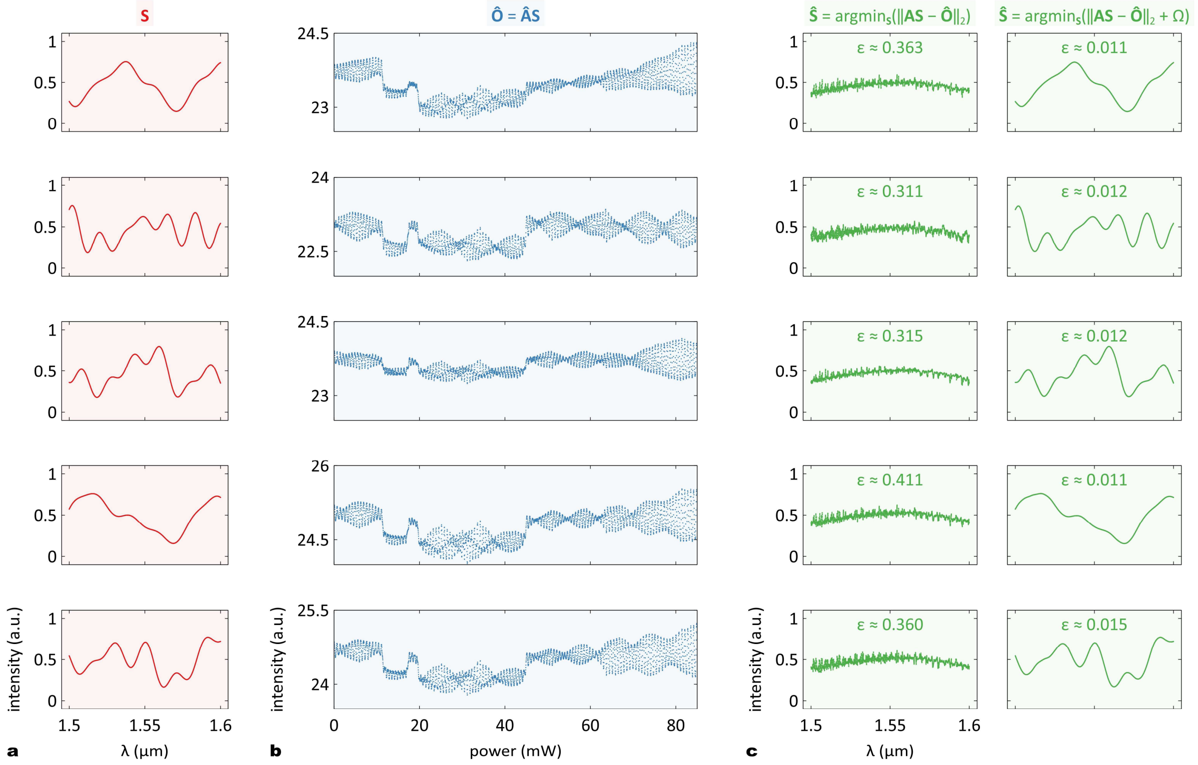

Fig. S13 Reconstruction of continuous spectra. (**a**) Testing input spectra (**S**). (**b**) Calculated output signals (**Ô**). (**c**) Reconstructed spectra (**Ŝ**) with relative errors (ε) labeled.

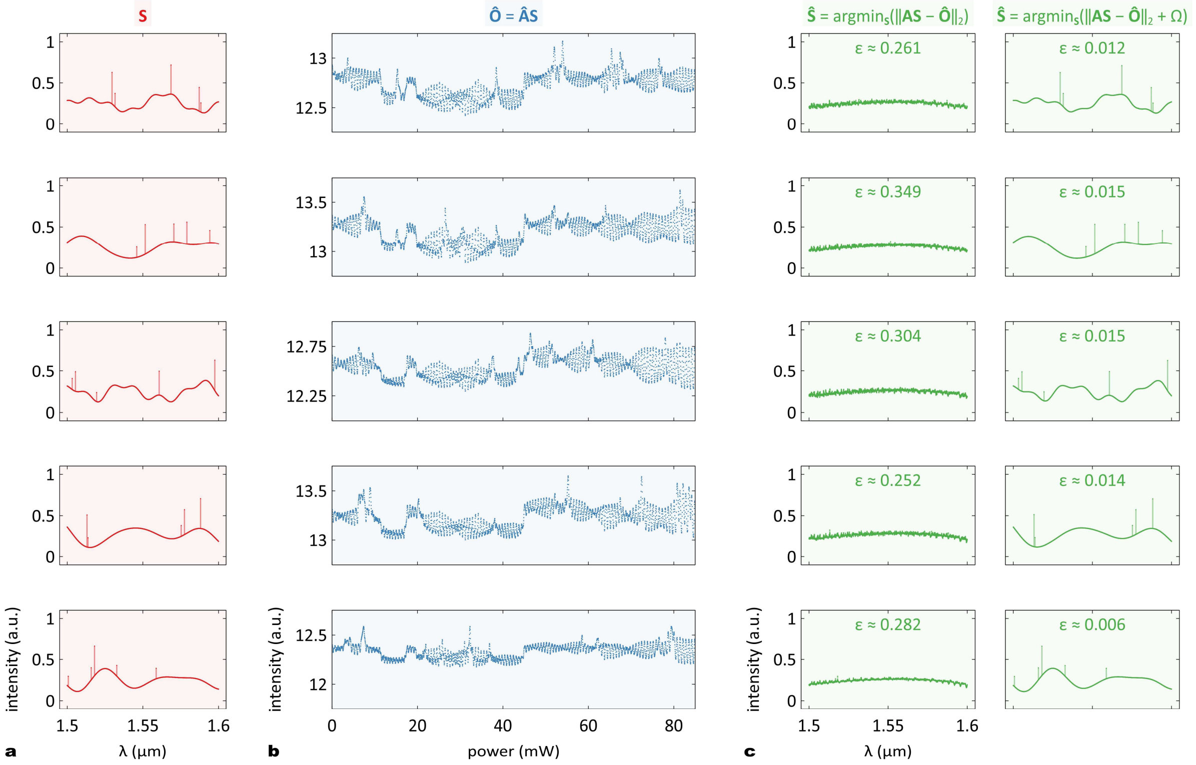

Fig. S14 Reconstruction of hybrid spectra. (**a**) Testing input spectra (**S**). (**b**) Calculated output signals (**Ô**). (**c**) Reconstructed spectra (**Ŝ**) with relative errors (ε) labeled.

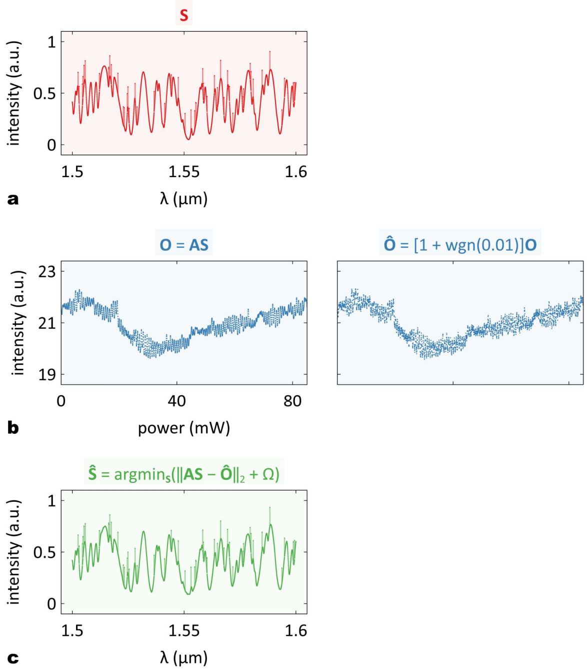

Fig. S15 Reconstruction example for a more complex spectrum. (**a**) Testing input spectrum (**S**). (**b**) Left penal: calculated output signal (**O**). Right panel: output signal (**Ô**) with a white Gaussian noise imposed. Here, *wgn*(·) denotes the white Gaussian noise function. The noise strength is set as 0.01. (**c**) Reconstructed spectrum (**Ŝ**). Here, we present a numerical example to show the feasibility of reconstruction for more complex spectra. The testing **S** has both continuous and discrete features. The continuous part of **S** contains substantial high-frequency components that are completely stochastic, whereas the discrete part of **S** involves 50 spectral lines with fully randomized positions and amplitudes. Moreover, a strong white Gaussian noise [*wgn*(0.01)] is imposed onto **Ô** to further prove the robustness against environmental perturbations. It should be

noted that this noise level (1%, or −20 dB) is much higher than that in the measurement system. The hyperparameters in penalty terms (i.e., ζ_1 and ζ_2) are not preset to specific values and are optimized solely by the cross validation (CV). From Fig. S15(c), the complex spectrum can be precisely reconstructed with low errors of $\varepsilon \approx 0.030$, even in the presence of strong noises. Such reconstruction capability is guaranteed by the sufficient decorrelation of wavelength channels. To be specific, the Picard condition can always be fulfilled for arbitrary types of **S** (see Section S7), indicating that a convergent and stable solution can always be obtained through iterative optimization when the penalty terms are properly chosen, and the noise perturbation is compensated.

S11. Additional information about the calibration process

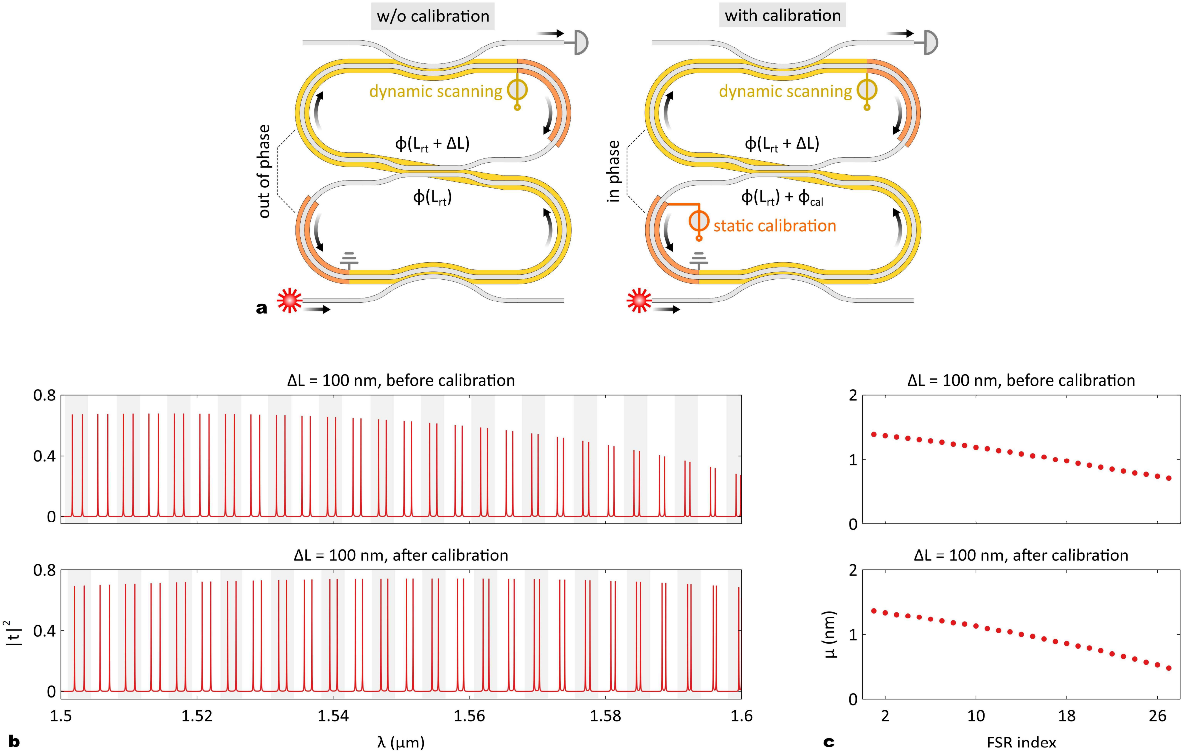

Fig. S16 Calibration process. (**a**) Illustration of the calibration process. The photonic molecule could be out of phase due to fabrication flaws. Here, it is assumed that the round-trip lengths (L_{rt}) are slightly different between two resonators, i.e., $\Phi(L_{rt}) \neq \Phi(L_{rt} + \Delta L)$. In our design, there are two extra segments of heaters (i.e., calibration regions) that can be exploited to offset the phase deviation, i.e., $\Phi(L_{rt}) + \Phi_{cal} = \Phi(L_{rt} + \Delta L)$, during power scanning. Calculated (**b**) transmission responses ($|t|^2$) and (**c**) splitting strengths (μ) before and after calibration. The L_{rt} difference is set as $\delta L = 100$ nm, as an example. Before calibration, the peak value of $|t|^2$ drops at longer wavelengths. The dispersion of μ is also diminished. After calibration, the uniform $|t|^2$ and dispersive μ are restored. This configuration improves the reliability and reproducibility of the proposed spectrometer. For the fabricated device, the measured $|t|^2$ is virtually uniform over all resonant peaks, indicating that the photonic molecule is already in-phase. Therefore, the calibration was not conducted in the experiment.

S12. Characterization of dispersion-engineered couplers

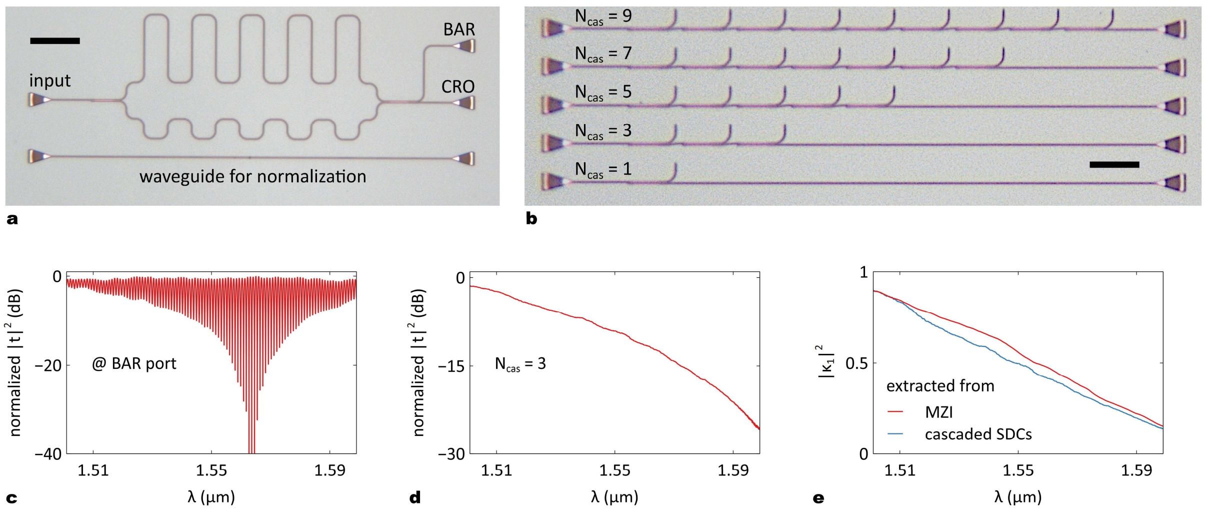

Fig. S17 Straight directional couplers (SDC). (**a**) Microscope image of the fabricated testing Mach-Zehnder interferometer (MZI). The scale bar represents 60 μm. (**b**) Microscope image of the fabricated cascading structures with N_{cas} identical SDCs. The scale bar represents 40 μm. (**c**) Normalized MZI transmission response ($|t|^2$). (**d**) Normalized $|t|^2$ with $N_{cas} = 3$. (**e**) Extracted coupling strengths ($|\kappa_1|^2$) at varying wavelengths.

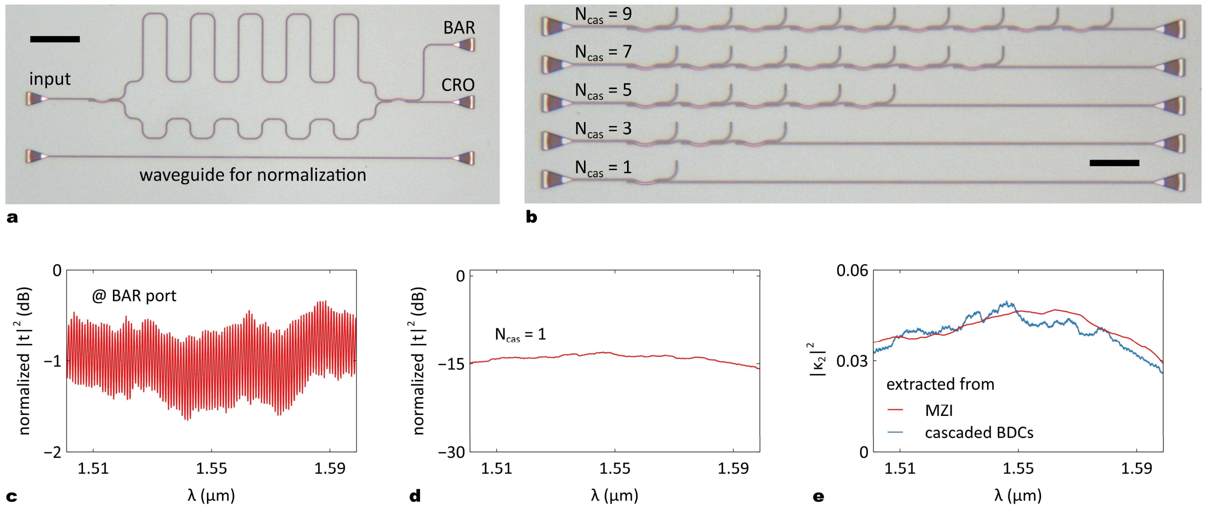

Fig. S18 Bent directional couplers (BDC). (**a**) Microscope image of the fabricated testing Mach-Zehnder interferometer (MZI). The scale bar represents 60 μm. (**b**) Microscope image of the fabricated cascading structures with N_{cas} identical BDCs. The scale bar represents 40 μm. (**c**) Normalized MZI transmission response ($|t|^2$). (**d**) Normalized $|t|^2$ with $N_{cas} = 1$. (**e**) Extracted coupling strengths ($|\kappa_2|^2$) at varying wavelengths.

Two sets of testing structures, i.e., a Mach-Zehnder interferometer (MZI) and cascading structures, were fabricated on the same chip to characterize the dispersion of straight directional couplers (SDC), as shown in Figs. S17(a-b). The fabricated MZI is formed by a 600-μm phase shifter and a pair of SDCs at both ends. Figure S17(c) shows the normalized transmission response ($|t|^2$) at the BAR port. The extinction ratio (ER) varies significantly over a 100-nm bandwidth, indicating a strong dispersion of coupling strengths ($|\kappa_1|^2$). The following equation is used to extract $|\kappa_1|^2$ from ERs[S6]:

$$\left|\kappa_1\right|^2 = \frac{1}{2} \pm \frac{1}{2}\sqrt{\frac{1}{10^{\mathrm{ER}}}}. \tag{S10}$$

The obtained $|\kappa_1|^2$ is shown in Fig. S17(e). In Fig. S17(d), we present the measured $|t|^2$ for the cascaded SDCs with $N_{cas} = 3$. $|\kappa_1|^2$ of a single SDC can be derived by averaging the response, as shown in Fig. S17(e). As a confirmation, $|\kappa_1|^2$ obtained from the MZI and cascaded SDCs are highly comparable. The measured coupling-strength variation is $|\kappa_1|^2 \in [0.12, 0.90]$, which agrees well with simulations. The same characterization method was also applied to measure the dispersion property of bent directional couplers (BDC), as shown in Fig. S18. The measured coupling strength is $|\kappa_2|^2 \approx 0.045$ at $\lambda = 1.55$ μm. Over a 100-nm bandwidth, a weak variation of $|\kappa_2|^2$ can be clearly observed.

S13. Analysis of noise blurring

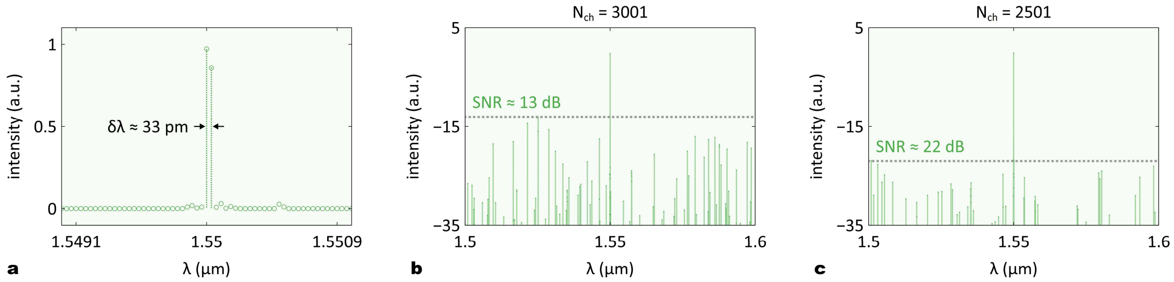

Fig. S19 Signal-to-noise ratio analysis. Experimental reconstruction of (**a**) dual spectral lines with N_{ch} = 3001 and a single spectral line with (**b**) N_{ch} = 3001 and (**c**) N_{ch} = 2501.

According to the measured correlation function shown in Fig. 6(e), the theoretical resolution limit of the fabricated spectrometer is δλ ≈ FWHM_C < 33 pm, with the corresponding channel number of N_{ch} = 3001. In Fig. S19(a), we present the experimental reconstruction result for dual spectral lines with a spacing of ≈ 33 pm. The peaks can be clearly identified from the intentionally deviated intensities [see also Fig. 7(c)], demonstrating that the δλ limit can be reached. Figure S19(b) shows the reconstructed single spectral line at λ = 1.55 μm. The signal-to-noise ratio is measured to be SNR ≈ 13 dB. The relatively low SNR originates in the noise blurring effect shown in Fig. 6(f). To be specific, the measured transmission matrix (**A**) also involves noises (Δ**A**), i.e., $\mathbf{A} = \mathbf{A}_{ext} + \Delta\mathbf{A}$. Here, $\mathbf{A}_{\mathrm{ext}}$ denotes the exact form of **A**. Considering the errors in both **A** and recorded signal ($\hat{\mathbf{O}}$), Eq. (9) is rewritten as:

$$\hat{\mathbf{S}} = \arg\min_{\mathbf{S}}\left(\left\|\mathbf{A}_{ext}\mathbf{S} - \mathbf{O} + (\Delta\mathbf{A}\mathbf{S} - \mathbf{e})\right\|_2 + \Omega\right). \tag{S11}$$

Notably, the Δ**AS** term will change at each iteration when **S** is updated, making it more difficult to find the global optimum. A straightforward solution to this issue is to slightly reduce N_{ch} since the blurring only occurs at higher vector indices and the number of blurred vectors will diminish under a smaller N_{ch}. The reconstruction result with N_{ch} = 2501 is shown in Fig. S19(c). The improved signal-to-noise ratio is SNR ≈ 22 dB. At different wavelengths, a high SNR ≈ 20 ~ 25 dB can be achieved, which is comparable to the previously reported results[S7,S8]. In short, the resolution of δλ ≈ 33 pm can only be accomplished at the cost of a lower SNR. To be rigorous, under the available measurement accuracy, the truly accessible resolution is characterized to be δλ = 40 pm. To improve the attainable δλ, it is essential to further lower the noise in the measured **A**. By applying the pre-treatments discussed in Section S5, most high-frequency noises, e.g., the Johnson noise of the optical power meter, can be filtered out; hence, Δ**A** is governed by lower-frequency noises caused by environmental perturbations, e.g., the vibration at the fiber-chip interface and temperature fluctuations, with their intrinsic frequencies much lower than the sampling frequency of wavelength sweeping. The mechanical vibration can be prevented by gluing the input fiber with epoxy and using

monolithically integrated photodetector. The temperature sensitivity can be reduced by utilizing material platforms with lower thermo-optical coefficients, such as silicon nitride.

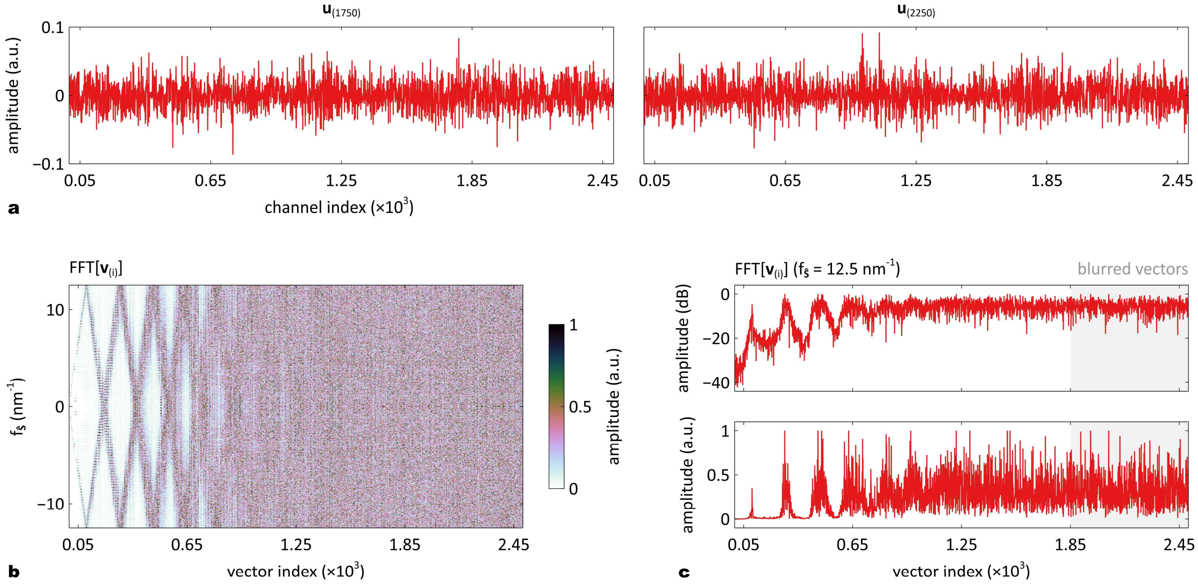

Fig. S20 Noise-blurring analysis. (**a**) 1750-th and 2250-th left singular vectors [$\mathbf{u}_{(1750)}$, $\mathbf{u}_{(2250)}$]. (**b**) Fast Fourier transform (FFT) result for right singular vectors [$\mathbf{v}_{(i)}$]. (**c**) FFT result for the highest frequency component of $\hat{\mathbf{S}}$ at the linear (lower panel) and logarithmic (upper panel) scales.

One might find that the blurred vectors are not entirely truncated with $N_{ch} = 2501$. Here, we give an explanation as to why the resolution of $\delta\lambda = \mathrm{BW}/(N_{ch} - 1)$ can be attained even with blurring. Figure S20(a) shows an unaffected $\mathbf{u}_{(1750)}$ and a blurred $\mathbf{u}_{(2250)}$. In contrast to the pulse-like function shown in Figs. 4(h) and S8(b), $\mathbf{u}_{(2250)}$ has a uniform envelope just like $\mathbf{u}_{(1750)}$. Furthermore, the sampling trajectory shown in Fig. 6(f) can be clearly distinguished from the noise background, demonstrating that these blurred $\mathbf{u}_{(i)}$ still offer sampling and are still usable. Fourier analysis is then performed on the right singular vectors [$\mathbf{v}_{(i)}$] of the measured $\mathbf{A}$, as shown in Fig. S20(b). As aforementioned, each $\mathbf{v}_{(i)}$ is a constitution basis of the reconstructed spectrum ($\hat{\mathbf{S}}$). Unlike $\mathbf{u}_{(i)}$, $\mathbf{v}_{(i)}$ is disordered in the Fourier domain. In the FFT map, the highest FFT frequency of $\hat{\mathbf{S}}$ (denoted as $f_{\hat{S}}$) corresponds to the resolution gird of $\mathbf{A}$:

$$\max\left(f_{\hat{S}}\right) = \frac{1}{2\delta\lambda}. \tag{S12}$$

Figure S20(c) shows the calculated FFT[$\mathbf{v}_{(i)}$] at the highest $f_{\hat{S}} = 12.5\ \mathrm{nm}^{-1}$. Remarkably, those $\mathbf{v}_{(i)}$ with lower vector indices already involve a large fraction of the highest frequency components of $\hat{\mathbf{S}}$. In other words, those unaffected $\mathbf{u}_{(i)}$ already collect most essential information for the reconstruction of $\hat{\mathbf{S}}$ in the finest detail. Also, by checking with the Picard plots shown Figs. 6(h) and S9, it is confirmed that the linear inverse problem always has a convergent and stable solution.

S14. Additional information about the monolithic measurement system

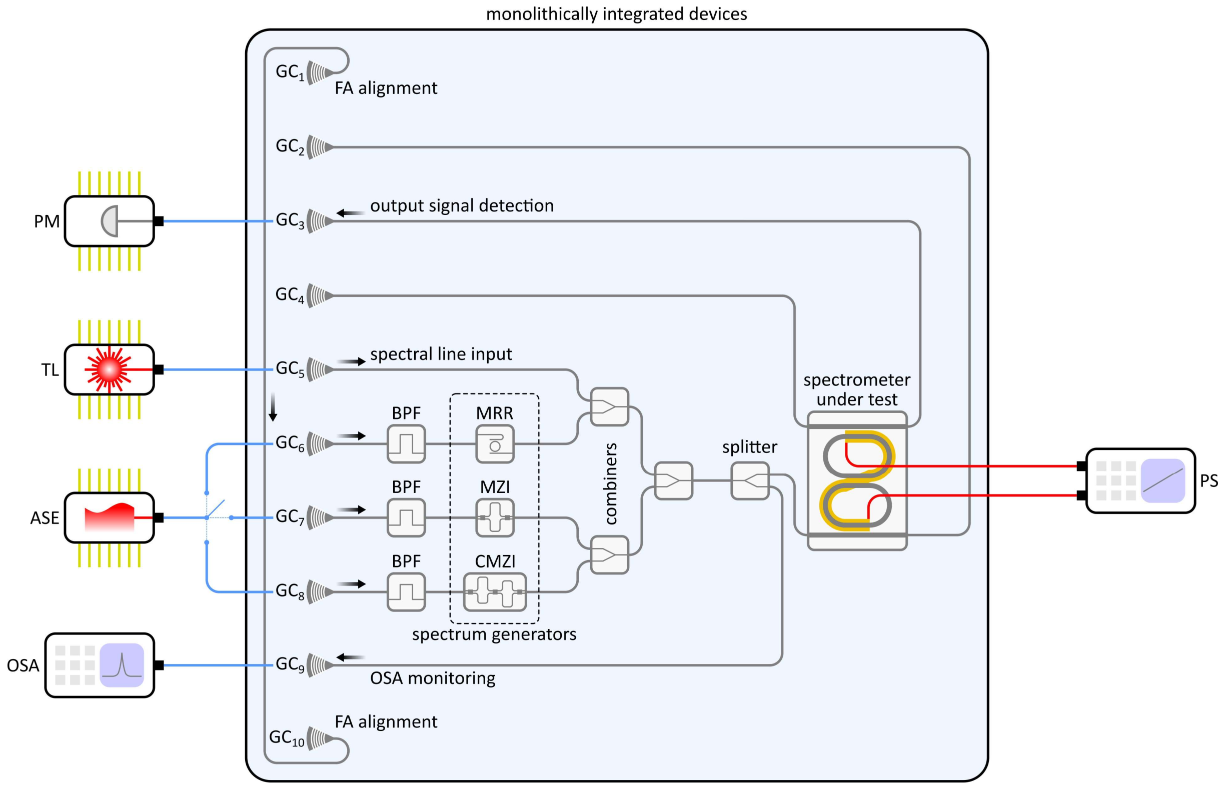

Fig. S21 Monolithic measurement system. The shaded box contains all the monolithically integrated devices. The blue lines represent fiber connections. The red lines represent electric connections. The gray lines represent SOI waveguides. FA, fiber array. BPF, bandpass filter. MRR, micro-ring resonator. MZI, Mach-Zehnder interferometer. CMZI, cascaded MZIs. GC, grating coupler. PM, optical power meter. PS, electric power source. TL, tunable laser. ASE, amplified spontaneous emission source. OSA, optical spectrum analyzer.

In this work, we propose to use a monolithic measurement system to implement the spectrum reconstruction, as illustrated in Fig. S21. An array of grating couplers (denoted as $GC_{1\text{-}10}$) was employed as an interface between on-chip devices and a fiber array (FA). GC_1 and GC_{10} were located at two lateral sides for alignment purposes. $GC_{5\text{-}8}$ are input ports where incident light was launched. GC_2 and GC_4 are dummy ports that are left unused. A tunable laser (TL, Keysight 8164B) was connected to GC_5 to produce single spectral lines at different wavelengths. This port was also utilized to measure the transmission matrix. Dual spectral lines were produced by adopting a second TL (Keysight 8163B) with a fiber 3-dB coupler (not displayed in Fig. S21), whose insertion loss was compensated by increasing the lasing power. An amplified spontaneous emission source (ASE, FiberLake) was connected to $GC_{6\text{-}8}$ to produce continuous spectra. These ports were routed to three different types of integrated filters, i.e., the micro-ring resonator (MRR), Mach-Zehnder interferometer (MZI), and cascaded MZIs (CMZI), in order to load various spectral details onto the emission spectrum of ASE. The ASE emission covers the wavelength range from λ = 1.52 μm to 1.62 μm, which slightly exceeds the target bandwidth. The out-of-range emission was eliminated by integrated bandpass filters (BPF). The spectral envelope shown in see Figs. 7(d-e) mainly results from the non-

uniform output of ASE and filtering of BPF. Four light paths were converged to a single bus, which was then split and routed to the spectrometer under test and GC_9. As a reference, the output at GC_9 was monitored by an optical spectrum analyzer (OSA, Yokogawa AQ6370D). The raw data from OSA, which have a fine resolution of 20 pm, are resampled to produce the reference spectra shown in Figs. 7(b-e). The insertion losses of GCs and integrated couplers are also subtracted from the displayed spectra. The output signal was captured by an optical power meter (PM, Agilent 81532A) that was connected to GC_3. A programmable electric power source (PS, Keithley 2400) was used to manage the power scanning. Data acquisition was implemented through a 16-bit analog-to-digital converter (ADC, NI USB-6361). The sampling time is ≈ 0.3 s at each scanning step. It is feasible to accelerate the acquisition even further by replacing the PM with a high-speed photodetector. The chip was mounted on a commercial thermo-electric cooler (TEC) to stabilize the ambient temperature.

S15. Additional experimental reconstruction results for single spectral lines

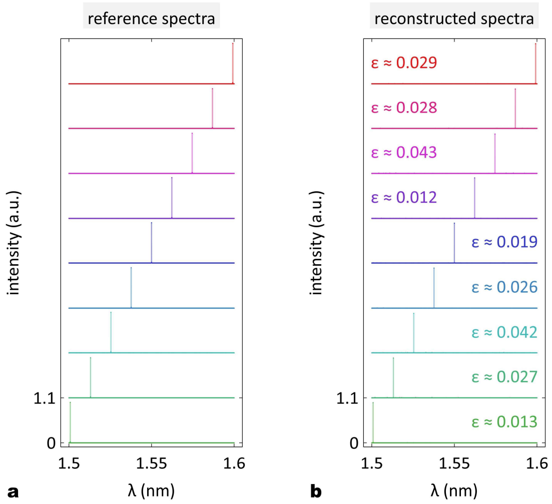

Fig. S22 Experimental reconstruction of single spectral lines. (**a**) Reference spectra. (**b**) Reconstructed spectra with relative errors (ε) labeled.

S16. Performance comparison of reported integrated spectrometers

Table S1. Performance comparison of reported integrated spectrometers

Type	Footprint [μm^2]	Δλ [pm]	BW [nm]	P_{max} [mW]	N_{ch}	N_0	N_{ch}/N_0	RFP [$pm \cdot mm^2$]	BFR [$nm \cdot \mu m^{-2}$]	CFR [μm^{-2}][d]
AWG[S9]	8000×8000	150	7.5	/	50	50	1	9600	1.2×10^{-7}	7.8×10^{-7}
EDG[S10]	6000×9000	500	30	/	60	60	1	2.7×10^{4}	5.6×10^{-7}	1.1×10^{-6}
EDG[S11]	2×10^{12}	150	148	/	926	926	1	3×10^{8}	7.4×10^{-11}	4.6×10^{-10}
MRR[S12]	1×10^{6}	600	50	/	81[b]	81	1	600	5×10^{-5}	8.1×10^{-4}
FTS[S13]	NM[a]	50	0.78	/	16	32	0.5	/	/	/
FTS[S14]	1.2×10^{7}	40	0.75	/	16	32	0.5	480	6.3×10^{-8}	1.3×10^{-6}
FTS[S15]	1.2×10^{8}	6000	600	/	100	200	0.5	7.2×10^{5}	5×10^{-6}	8.3×10^{-7}
FTS[S16]	320×410	≈ 40	≈ 0.4	/	8	16	0.5	5.25	3.1×10^{-6}	6.1×10^{-5}
FTS[S17]	3×10^{6}	5000	400	/	81	32	2.5	1.5×10^{4}	1.3×10^{-4}	2.7×10^{-5}
FTS[S18]	22×512	4000	96	/	25[b]	CCD[c]	/	45.1	8.5×10^{-3}	2.2×10^{-3}
FTS[S19]	1×10^{5}	6000	100	/	17[b]	CCD[c]	/	600	1×10^{-3}	1.7×10^{-4}
FTS[S20]	1×10^{7}	≈ 5000	500	NM[a]	101[b]	CCD[c]	/	5×10^{4}	1×10^{-3}	1.0×10^{-5}
SS[S7]	100×50	600	25	/	42[b]	25	1.7	3	5×10^{-3}	8.4×10^{-3}
SS[S21]	200×50	3400	40	/	13	13	1	34	4×10^{-3}	1.3×10^{-3}
SS[S22]	12.8×30	250	30		121[b]	8	15.1	0.096	0.078	0.32
SS[S23]	500×500	10	2	/	332	40	8.3	2.5	8×10^{-6}	1.3×10^{-3}
SS[S24]	1600×2100	16	2	/	126[b]	12	10.5	53.8	5.9×10^{-7}	3.8×10^{-5}
SS[S25]	8500×8500	1	12	/	$>1\times10^{4}$	CCD[c]	/	72.3	1.7×10^{-7}	1.4×10^{-4}
SS[S26]	NM[a]	100	6.3	/	64	4	16	/	/	/
SS[S27]	35×260	450	180	/	401[b]	32	12.5	4.1	0.019	0.044
SS[S28]	220×520	20	12	/	600	64	9.4	2.3	1.1×10^{-4}	5.2×10^{-3}
AWG/MRR[S29]	3000×3000	100	25.4	30×9	255	9	28	900	2.8×10^{-6}	2.8×10^{-5}
MRR[S30]	3.5×10^{5}	5	10	50×10	1941[b]	10	194	1.75	2.9×10^{-5}	5.5×10^{-3}
AWG/MRR[S31]	1150×1250	750	57.5	/	70	70	1	1100	4×10^{-5}	4.9×10^{-5}
AWG/MRR[S32]	200×270	200	70	NM[a]	350	10	35	10.8	1.3×10^{-3}	6.5×10^{-3}
Nanobeam[S8]	18×250	160	16	30×3	101[b]	3	34	0.72	3.6×10^{-3}	0.022
Grating[S33]	3.6×10^{4}	510	102.7	873	201	7	29	18.4	2.9×10^{-3}	5.6×10^{-3}
Nanobeam[S34]	6×111	1000	35	/	38	38	1	0.67	0.053	0.057
FTS[S35]	NM[a]	200	20	99	101[b]	1	64	/	/	/
FTS[S36]	1×10^{6}	3000	56	5100	19[b]	1	19	3000	5.6×10^{-5}	1.9×10^{-5}
FTS[S37]	NM[a]	470	90	1500	192[b]	1	192	/	/	/
FTS[S38]	NM[a]	160	180	5000	1126	1	1126	/	/	/
**This work**	**60×60**	**40**	**100**	**75**	**2501**	**1**	**2501**	**0.14**	**0.028**	**0.69**

AWG, arrayed waveguide grating.

EDG, echelle diffraction grating.

MRR, micro-ring resonator.

FTS, Fourier transform spectrometer.

SS, speckle spectrometer.

$\Delta\lambda$, spectral resolution.

BW, working bandwidth.

P_{max}, maximum electric power applied.

N_{ch}, number of wavelength channels.

N_0, number of spatial channels.

RFP, resolution-footprint product.

BFR, bandwidth-to-footprint ratio.

CFR, channel-to-footprint ratio.

[a]Not mentioned in the paper.

[b]Derived from the claimed $\Delta\lambda$ and BW.

[c]Measured with CCD.

[d]Some applications need the simultaneous detection of multiple wavelength channels, for which CFR may not be a suitable figure of merit.

References

S1. Réfrégier P. *Noise theory and application to physics: from fluctuations to information*. Springer Science & Business Media (2004).

S2. Hansen PC. The discrete Picard condition for discrete ill-posed problems. *BIT Numerical Mathematics* **30**, 658-672 (1990).

S3. IEEE standard specification format guide and test procedure for single-axis interferometric fiber optic gyros. *IEEE Std 952-1997*, 1-84 (1998).

S4. Golub GH, Heath M, Wahba G. Generalized cross-validation as a method for choosing a good ridge parameter. *Technometrics* **21**, 215-223 (1979).

S5. Achim A, Christian BH, Mark ES. lassopack: Model selection and prediction with regularized regression in Stata. *The Stata Journal* **20**, 176-235 (2020).

S6. Wang Y*, et al.* Polarization-independent mode-evolution-based coupler for the silicon-on-insulator platform. *IEEE Photon J* **10**, 1-10 (2018).

S7. Redding B, Liew SF, Sarma R, Cao H. Compact spectrometer based on a disordered photonic chip. *Nat Photon* **7**, 746-751 (2013).

S8. Zhang J, Cheng Z, Dong J, Zhang X. Cascaded nanobeam spectrometer with high resolution and scalability. *Optica* **9**, 517 (2022).

S9. Cheben P*, et al.* A high-resolution silicon-on-insulator arrayed waveguide grating microspectrometer with sub-micrometer aperture waveguides. *Opt Express* **15**, 2299-2306 (2007).

S10. Xiao M, Mingyu L, Jian-Jun H. CMOS-compatible integrated spectrometer based on Echelle diffraction grating and MSM photodetector array. *IEEE Photon J* **5**, 6600807-6600807 (2013).

S11. Calafiore G*, et al.* Holographic planar lightwave circuit for on-chip spectroscopy. *Light Sci Appl* **3**, e203-e203 (2014).

S12. Xia Z*, et al.* High resolution on-chip spectroscopy based on miniaturized microdonut resonators. *Opt Express* **19**, 12356-12364 (2011).

S13. Bock PJ*, et al.* Subwavelength grating Fourier-transform interferometer array in silicon-on-insulator. *Laser Photon Rev* **7**, L67-L70 (2013).

S14. Velasco AV*, et al.* High-resolution Fourier-transform spectrometer chip with microphotonic silicon spiral waveguides. *Opt Lett* **38**, 706-708 (2013).

S15. Yang M, Li M, He JJ. Static FT imaging spectrometer based on a modified waveguide MZI array. *Opt Lett*

42, 2675-2678 (2017).

S16. Gonzalez-Andrade D*, et al.* Broadband Fourier-transform silicon nitride spectrometer with wide-area multiaperture input. *Opt Lett* **46**, 4021-4024 (2021).

S17. Yoo KM, Chen RT. Dual-polarization bandwidth-bridged bandpass sampling Fourier transform spectrometer from visible to near-infrared on a silicon nitride platform. *ACS Photon* **9**, 2691-2701 (2022).

S18. le Coarer E*, et al.* Wavelength-scale stationary-wave integrated Fourier-transform spectrometry. *Nat Photon* **1**, 473-478 (2007).

S19. Nie X, Ryckeboer E, Roelkens G, Baets R. CMOS-compatible broadband co-propagative stationary Fourier transform spectrometer integrated on a silicon nitride photonics platform. *Opt Express* **25**, A409-A418 (2017).

S20. Pohl D*, et al.* An integrated broadband spectrometer on thin-film lithium niobate. *Nat Photon* **14**, 24-29 (2019).

S21. Hartmann W*, et al.* Waveguide-integrated broadband spectrometer based on tailored disorder. *Advanced Optical Materials* **8**, 1901602 (2020).

S22. Hadibrata W, Noh H, Wei H, Krishnaswamy S, Aydin K. Compact, high-resolution inverse-designed on-chip spectrometer based on tailored disorder modes. *Laser Photon Rev* **15**, 2000556 (2021).

S23. Redding B, Fatt Liew S, Bromberg Y, Sarma R, Cao H. Evanescently coupled multimode spiral spectrometer. *Optica* **3**, 956 (2016).

S24. Piels M, Zibar D. Compact silicon multimode waveguide spectrometer with enhanced bandwidth. *Sci Rep* **7**, 43454 (2017).

S25. Paudel U, Rose T. Ultra-high resolution and broadband chip-scale speckle enhanced Fourier-transform spectrometer. *Opt Express* **28**, 16469-16485 (2020).

S26. Yi D, Zhang Y, Wu X, Tsang HK. Integrated multimode waveguide with photonic lantern for speckle spectroscopy. *IEEE J Quant Electron* **57**, 1-8 (2021).

S27. Li A, Fainman Y. On-chip spectrometers using stratified waveguide filters. *Nat Commun* **12**, 2704 (2021).

S28. Zhang Z, Li Y, Wang Y, Yu Z, Sun X, Tsang HK. Compact high resolution speckle spectrometer by using linear coherent integrated network on silicon nitride platform at 776 nm. *Laser Photon Rev* **15**, 2100039 (2021).

S29. Zheng S*, et al.* A single-chip integrated spectrometer via tunable microring resonator array. *IEEE Photon J* **11**, 1-9 (2019).

S30. Zhang L, Zhang M, Chen T, Liu D, Hong S, Dai D. Ultrahigh-resolution on-chip spectrometer with silicon photonic resonators. *Opto-Electronic Advances* **5**, 210100-210100 (2020).

S31. Zhang Z, Wang Y, Tsang HK. Tandem configuration of microrings and arrayed waveguide gratings for a high-resolution and broadband stationary optical spectrometer at 860 nm. *ACS Photon* **8**, 1251-1257 (2021).

S32. Zhang Z*, et al.* Integrated scanning spectrometer with a tunable micro-ring resonator and an arrayed waveguide grating. *Photon Res* **10**, A74 (2022).

S33. Sun C*, et al.* Broadband and high-resolution integrated spectrometer based on a tunable FSR-free optical filter array. *ACS Photon*, in press (2022).

S34. Cheng Z*, et al.* Generalized modular spectrometers combining a compact nanobeam microcavity and computational reconstruction. *ACS Photon* **9**, 74-81 (2021).

S35. Kita DM*, et al.* High-performance and scalable on-chip digital Fourier transform spectroscopy. *Nat Commun* **9**, 4405 (2018).

S36. Souza M, Grieco A, Frateschi NC, Fainman Y. Fourier transform spectrometer on silicon with thermo-optic non-linearity and dispersion correction. *Nat Commun* **9**, 665 (2018).

S37. Zheng SN*, et al.* Microring resonator-assisted Fourier transform spectrometer with enhanced resolution and large bandwidth in single chip solution. *Nat Commun* **10**, 2349 (2019).

S38. Li A, Fainman Y. Integrated slicon Fourier transform spectrometer with broad bandwidth and ultra-high resolution. *Laser Photon Rev* **15**, 2000358 (2021).
